# A *Drosophila* RNAi screen reveals conserved glioblastoma-related adhesion genes that regulate collective cell migration

**DOI:** 10.1093/g3journal/jkab356

**Published:** 2021-10-11

**Authors:** Nirupama Kotian, Katie M Troike, Kristen N Curran, Justin D Lathia, Jocelyn A McDonald

**Affiliations:** 1 Division of Biology, Kansas State University, Manhattan, KS 66506, USA; 2 Department of Cardiovascular and Metabolic Sciences, Lerner Research Institute, Cleveland Clinic, Cleveland, OH 44195, USA

**Keywords:** *Drosophila*, cell adhesion, collective migration, glioblastoma, *α-catenin*, *Symplekin*, *Lachesin*, *roughest*, *dreadlocks*, *Wnt4*, *dachsous*, *fat*

## Abstract

Migrating cell collectives are key to embryonic development but also contribute to invasion and metastasis of a variety of cancers. Cell collectives can invade deep into tissues, leading to tumor progression and resistance to therapies. Collective cell invasion is also observed in the lethal brain tumor glioblastoma (GBM), which infiltrates the surrounding brain parenchyma leading to tumor growth and poor patient outcomes. *Drosophila* border cells, which migrate as a small cell cluster in the developing ovary, are a well-studied and genetically accessible model used to identify general mechanisms that control collective cell migration within native tissue environments. Most cell collectives remain cohesive through a variety of cell–cell adhesion proteins during their migration through tissues and organs. In this study, we first identified cell adhesion, cell matrix, cell junction, and associated regulatory genes that are expressed in human brain tumors. We performed RNAi knockdown of the *Drosophila* orthologs in border cells to evaluate if migration and/or cohesion of the cluster was impaired. From this screen, we identified eight adhesion-related genes that disrupted border cell collective migration upon RNAi knockdown. Bioinformatics analyses further demonstrated that subsets of the orthologous genes were elevated in the margin and invasive edge of human GBM patient tumors. These data together show that conserved cell adhesion and adhesion regulatory proteins with potential roles in tumor invasion also modulate collective cell migration. This dual screening approach for adhesion genes linked to GBM and border cell migration thus may reveal conserved mechanisms that drive collective tumor cell invasion.

## Introduction

While migrating cells contribute to many processes during embryonic development and adult wound healing, abnormal cell migration drives tumor cell invasion and metastasis. During development and in cancer, cells either migrate as single cells or as interconnected small to large groups of cells called collectives (Friedl and Gilmour 2009; Friedl *et al.* 2012; Scarpa and Mayor 2016; Te Boekhorst *et al.* 2016b). Especially in cancer, cells can interconvert their modes of movement, transitioning from collective to single cell movement and back (Te Boekhorst and Friedl 2016a). A wide variety of cancer cells, including breast, colorectal, and thyroid carcinomas, are now known to migrate and invade as collectives both *in vitro* and *in vivo* (Cheung and Ewald 2016a; Wang *et al.* 2016; Kim *et al.* 2017; Ilina *et al.* 2018; Libanje *et al.* 2019; Padmanaban *et al.* 2019). Recent work has shown that tumor cell collectives promote tumor invasion and metastasis and may provide a mechanism for resistance to radiation (Aceto *et al.* 2014; Cheung *et al.* 2016b; Haeger *et al.* 2020).

**Figure 1:**
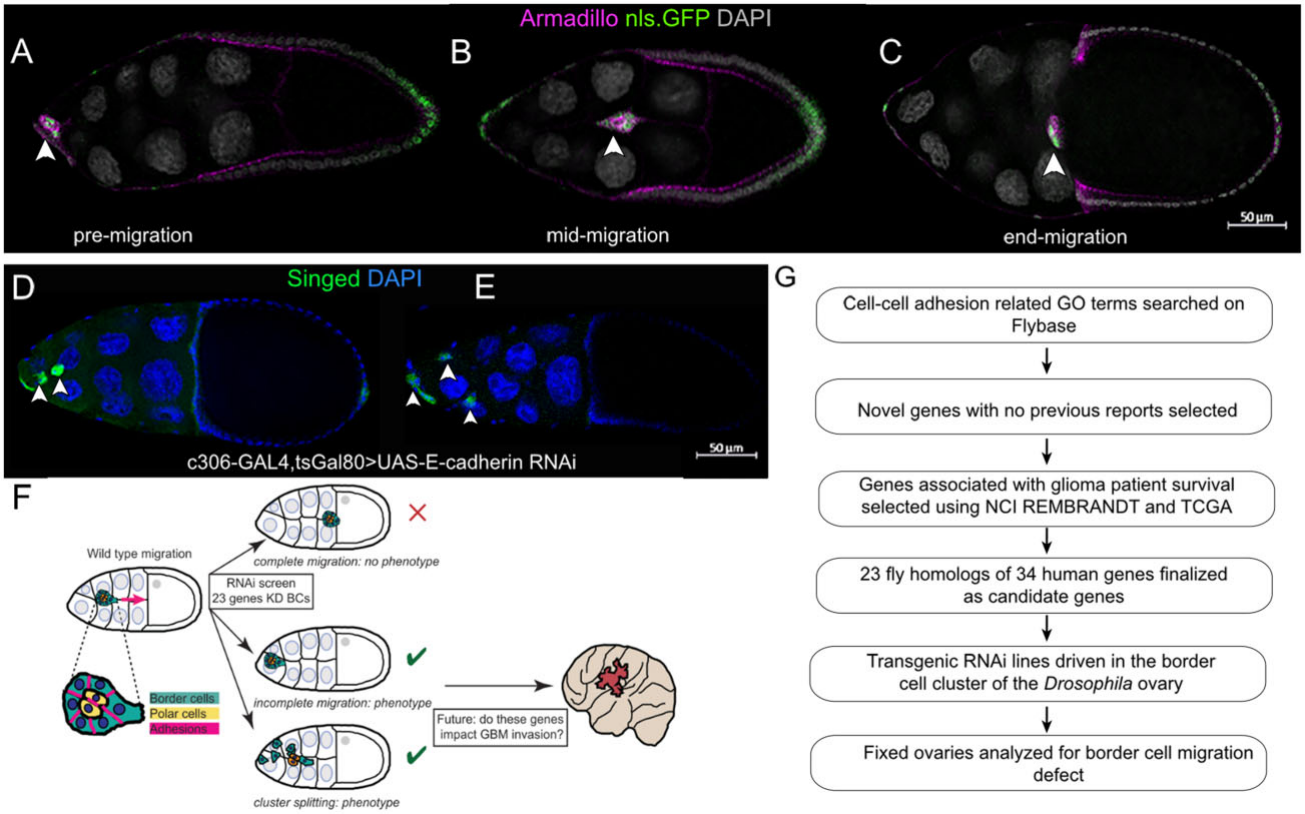
Screen to identify conserved GBM-associated adhesion genes in collective cell migration. (A–C) Migration of wild-type border cells in stages 9 and 10 egg chambers. *c306*-GAL4 drives nuclear GFP (UAS-nls.GFP, green) in egg chambers labeled with Armadillo (magenta) to show cell membranes, and DAPI to show nuclei (gray). Arrowheads indicate the position of the border cell cluster within the egg chamber during migration stages: pre-migration (A), mid-migration (B), and end-migration (C). (D, E) Knockdown of *E-cadherin* by RNAi (*c306*-GAL4 tsGAL80/+; +/UAS-E-cadherin RNAi line v103962) in border cells disrupts migration and cluster cohesion at stage 10. Arrowheads indicate border cell clusters and split clusters. (F) Schematic overview of the RNAi screening approach in border cells. (G) Experimental flow chart used to identify novel GBM-associated adhesion genes through *Drosophila* and human glioma databases.

The *Drosophila* border cells, which migrate collectively during late oogenesis, are a simple and genetically tractable model to identify genes required for collective cell migration (Montell *et al.* 2012; Saadin and Starz-Gaiano 2016). The border cell cluster consists of 4–8 epithelial-derived follicle cells that surround a central pair of polar cells (Figure  1, A–C and F). Individual border cells stay adhered together and their movement is coordinated as an entire unit during the 3- to 4-h journey to the oocyte (Figure  1, A–C). Multiple studies have used border cells to identify conserved genes that contribute to the migration of a variety of cancer cells, including those that invade as collectives (Yoshida *et al.* 2004; Madsen *et al.* 2015; Stuelten *et al.* 2018; Volovetz *et al.* 2020).

Glioblastoma (GBM) is the most common primary malignant brain tumor (Ostrom *et al.* 2014) and is refractory to many therapies including radiation and chemotherapy (Bao *et al.* 2006, Chen *et al.* 2012). Given the dismal prognosis of GBM, identifying the underlying mechanisms that drive progression, including cell invasion, remains an immediate priority. While many genes are known to be dysregulated in glioma patients, it is difficult to know which ones are most relevant to disease progression, including tumor invasion. We and others recently showed that glioma cells and GBM cancer stem cells (CSCs), which can drive tumor growth, migrate collectively in some contexts (Gritsenko *et al.* 2017; Gritsenko and Friedl 2018; Volovetz *et al.* 2020). Using several patient derived GBM CSC tumor models, we found that a gene required in border cells, the small GTPase Rap1, also contributes to GBM collective cell invasion (Chang *et al.* 2018; Sawant *et al.* 2018; Volovetz *et al.* 2020). Due to their cellular conservation and large degree of genetic homology with humans, *Drosophila* brain tumor models have been established and used to provide critical molecular insight into gliomas (Agnihotri *et al.* 2016; Chen *et al.* 2018; Chen and Read 2019; Chi *et al.* 2019; Gangwani *et al.* 2020). Because patient derived GBM CSC tumor models are less genetically accessible for screening approaches, and *Drosophila* glioma models entail multiple mutations, we turned to border cells as an initial simpler approach to identify conserved genes that may drive GBM collective tumor invasion but that may also have a more general role in collective cell migration.

Cell–cell and cell–matrix adhesions are critical for cells to stay together and move collectively *in vivo* (Friedl and Mayor 2017; Janiszewska *et al.* 2020). Thus, genes that regulate cell adhesion are strong candidates to promote collective cell cohesion, migration, and invasion. Here, we used the border cell system to screen a subset of adhesion and adhesion-related genes that have the potential to regulate GBM tumor migration and invasion. We selected conserved adhesion genes, genes associated with cell junctions, and genes that regulate cell–cell and cell–matrix adhesion. We further focused on those adhesion-related genes whose expression correlated with glioma patient survival but at the time of the screen did not have known functions in brain cancer. We performed an RNAi screen targeting 23 of these adhesion genes in border cells. Here, we report the identification of eight genes, *α-catenin* (*α-Cat*), *Symplekin* (*Sym*), *Lachesin* (*Lac*), *roughest* (*rst*), *dreadlocks* (*dock*), *Wnt4*, *dachsous* (*ds*), and *fat* (*ft*), whose knockdown disrupted border cell migration and/or cluster cohesion to differing degrees. We then identified three human orthologs of target genes that were enriched in the leading edge (LE) and invasive portion of GBM tumors, the α-Cat ortholog CTNNA2, the Lac ortholog NEGR1, and the Rst ortholog KIRREL3. While further work needs to be done to test these genes in GBM tumors, this study supports the use of *Drosophila* genetic approaches to provide insights into human diseases such as GBM.

## Materials and methods

### Identification of candidate genes

FlyBase FB2014_5 version (released September 9, 2014) was queried for adhesion genes using the following gene ontology (GO) controlled vocabulary (CV) terms: “apical junction complex,” “focal adhesion,” “cell adhesion molecule binding,” “cell junction maintenance,” “cell junction assembly,” and “cell–cell adherens junction.” A total of 133 *Drosophila* genes were identified. Human orthologs were identified by *Drosophila* RNAi Screening Center Integrative Ortholog Prediction Tool (DIOPT) scores (Hu *et al.* 2011; Table  1). A PubMed search was performed for these genes along with “glioma,” “GBM,” or “brain cancer” to eliminate genes with a known function in or association with these cancers. This step narrowed the list to 44 genes. The NCBI REMBRANDT database was next used to identify genes that are associated with brain cancer patient survival; these results were then confirmed using The Cancer Genome Atlas (TCGA). Genes associated with better (“positive”), or worse (“negative”) patient survival were selected. These analyses resulted in 23 conserved fly genes (34 human genes) that were the final candidate genes tested in the *in vivo* border cell RNAi screen.

**Table 1:** Drosophila and human brain tumor-associated adhesion genes

Gene name (Drosophila)	Human ortholog	DIOPT score out of 15	Best score	Best reverse score	Role in migration	Glioma patient survival	Role in glioma
alpha-Catenin	CTNNA1	12	No	Yes	Cytosolic αE-catenin pool sequestered to mitochondria of MDCK cells increases epithelial cell sheet migration but does not alter overall cadherin based adhesion^1^; loss leads to human keratinocyte cell migration^2^; anisotropically activated in epithelial sheet collective migration^3;^ collective cell migration in MDCK cells^4^; αE-catenin relocates to lamellipodia during migration of neural crest and glioma cells^5^; mouse angiogenic and glial cells^6,8^; homodimerize to PIP3 vesicles in lamellopodia^7^; prostate cancer cells^9^; wound healing in keratinocytes^10^; migration of dorsal ridge primordia^11^; epithelial invagination of *Drosophila* embryonic dorsal fold^12^	Negative	GBM cell migration, invasion, and proliferation *in vitro*^13,14^
	CTNNA2	13	Yes	Yes		ND	NA
	CTNNA3	7	No	Yes		Positive	NA
CAP	SORBS1	5	No	Yes	Links focal adhesion sites to nuclei during collective cardiac cell migration in flies^15,16^; ECM stiffness dependent mechanotransduction in mouse fibroblasts^17^	Positive	NA
Caskin	CASKIN1	4	Yes	Yes	NA	Positive	NA
	CASKIN2	4	Yes	Yes		Positive	NA
CG3770	LHFPL2	12	Yes	Yes	NA	Negative	NA
CG45049	PERP	10	No	Yes	NA	Negative	NA
Dachsous	DCHS1	11	Yes	Yes	Collective migration of larval epidermal cells^18,22^; glial migration during eye development^19,22^; collective tangential migration of murine facial motor neurons^20^; uniform axial orientation of *Drosophila* abdominal epithelial cells^21^	Negative	NA
Dock	NCK1	14	Yes	Yes	Dorsal appendage morphogenesis^23^; interacts with Misshapen during dorsal closure^24^; NCK1 promotes podosome biogenesis during tumor invasion^25^; endothelial front-rear polarity and migration^26^; formation of dorsal ruffles in mice embryonic fibroblasts^27^	Negative	Expression levels of NCK1 in gliomas^29^
Fat	FAT4	13	Yes	Yes	Collective tangential migration of murine facial motor neurons^19^; mutations in FAT4 causes defects in neuronal migration of cerebral organoids^28^	Positive	NA
G protein alpha i	GNAI2	11	No	Yes	Dorsal appendage morphogenesis^30^; modulates migration-proliferation dichotomy in breast and colon cancer cells^31^	Negative	Part of a signaling axis that enhances proliferation of GBM cells^32^
	GNAZ	5	No	Yes		Positive	NA
G protein alpha o	GNAI3	3	No	No	Promotes protrusion membrane dynamics^33^	Negative	NA
	GNAT3	3	No	No		Positive	NA
Gliotactin	NLGN2	5	Yes	No	Expressed in tricellular septate junctions in stage 10B egg chambers and border cells throughout migration^34^; Overexpression in the wing disc driven by apterous-GAL4 leads to migration of cells from dorsal to ventral compartment^35,36^	Positive	NA
Lachesin	LSAMP	5	No	No	Tracheal morphogenesis^37,38^	Positive	Expression of LSAMP negatively correlates with glioma survival in patients with EGFR mutation or amplification^39^
	NEGR1	9	Yes	Yes		Positive	NEGR1 variants and expression in pediatric gliomas^40^
Liprin-alpha	PPFIA1	11	No	Yes	Tumorigenesis and metastasis in fly eye tumor model ^41;^ metastasis of breast cancer cells in mice^42;^ regulates actin cytoskeleton through Rho-mDia pathway^43^; forms scaffold network that promote protrusion and FA turnover in motile and cancer cells^44,45^	Negative	PPFIA1 activation by PTPRD promotes glioma progression^46^
Lowfat	LIX1L	13	Yes	Yes	LIX1L is a driver of tumor growth and metastasis in hepatocellular carcinoma in mice^47^	Positive	NA
	LIX1	7	No	Yes		Positive	NA
Mesh	SUSD2	13	Yes	Yes	Promotes ovarian cancer metastasis^48^	Negative	SUSD2 is part of a signaling axis that contributes to glioma progression^49^
Parvin	PARVA	14	Yes	Yes	Invasion in human colorectal cancer cells, PARVB inhibits in vitro invasion of breast cancer cells^50,51^	Positive	NA
	PARVB	13	No	Yes		Positive	NA
Roughest	KIRREL1	13	Yes	Yes	KIRREL3 participates in myoblast directed migration^52^	Negative	NA
	KIRREL3	12	No	Yes		Negative	NA
	KIRREL2	11	No	Yes		Negative	NA
Schizo	IQSEC2	12	No	Yes	Cell movements during eye patterning^53^	Positive	NA
Shroom	SHROOM1	2	No	Yes	Regulates epithelial cell shape in the wing disc A-P boundary and required for tissue morphogenesis^54^; germband extension^55^; apical constriction during neural tube closure^56^; epithelial morphogenesis during axis elongation through actomyosin contractility^57^	Negative	NA
	SHROOM3	8	Yes	Yes		Negative	NA
Symplekin	SYMPK	14	Yes	Yes	Elevated Symplekin mRNA expression in human colorectal cancers including metastatic tumors^58^	Negative	NA
Vulcan	DLGAP1	7	Yes	Yes	Leg disc morphogenesis^59^	Positive	LncRNA upregulated in glioma correlates with poor prognosis^60,61^
	DLGAP2	6	No	Yes		Positive	NA
Wnt4	WNT9A	4	No	Yes	*Drosophila* salivary gland migration^63^; focal adhesion kinase regulation and cell migration during ovarian morphogenesis^63,64^; chick lung branching and development^65^	Positive	NA
Wunen	PLPP2	9	No	No	Caudal visceral mesoderm cell migration^66,67^; heart cell movement in flies^68^	Negative	NA

References are in Supplementary File S1.

ND, not determined; NA, not available.

### Bioinformatics analyses of human genes in tumor databases

Regional gene expression data from GBM tumor tissue was obtained from the Ivy Glioblastoma Atlas Project (Ivy GAP) database (https://glioblastoma.alleninstitute.org/static/home, accessed June 20, 2021), which contains gene expression data from several anatomical features of GBM tumors in a 41-patient dataset. Analysis of gene expression based on glioma grade (grades II, III, and IV) was performed using The Cancer Genome Atlas (TCGA) data downloaded from the Gliovis data portal (http://gliovis.bioinfo.cnio.es/, accessed May 5, 2021). The GEPIA (Gene Expression Profiling Interactive Analysis; http://gepia.cancer-pku.cn/, accessed March 30, 2021) database (Tang *et al.* 2017) was used to compare differential expression of gene orthologs in GBM tumor tissue (*n* = 163) and nontumor brain tissue (*n* = 207). Thresholds were set at a log2 fold change > 1 and a *P*-value < 0.01.

### *Drosophila* RNAi screen and genetics

All genetic crosses were set up at 25°C. The tub-GAL80ts (“tsGAL80”) transgene (McGuire *et al.* 2004) was included to prevent early GAL4-UAS expression and potential lethality at larval or pupal stages of development. *c306-*GAL4, tsGal80*;* Sco/CyO was used to drive UAS-RNAi line expression in border cells. UAS-mCherry RNAi crossed to c306-GAL4 tsGal80*;* Sco/CyO was used as a control. The expression pattern of *c306-*GAL4 was confirmed by crossing *c306-*GAL4, tsGal80*;* Sco/CyO to UAS-nls.GFP (BDSC 4776). Multiple RNAi lines for the 23 cell adhesion candidate genes and UAS-mCherry RNAi were obtained from the Vienna Drosophila RNAi Center (VDRC) or the Harvard Transgenic RNAi Project (TRiP) collection from the Bloomington Drosophila Stock Center (BDSC). All lines with stock numbers and construct IDs are listed in Table  2. Males from each UAS-RNAi line were crossed to virgin c*306-*GAL4, tsGal80 females. Three-to-five-day old F1 progeny females (*c306-*GAL4, tsGAL80/+; +/UAS-RNAi) from these crosses were fattened on wet yeast paste for ≥14 h at 29°C prior to dissection. This allowed maximum GAL4-UAS expression and full inactivation of tsGAL80. Each RNAi line was tested one time in the primary screen, with a subset of lines tested at least three times in the secondary screen unless otherwise noted (Table  2).

**Table 2:** Results of the border cell RNAi screen

Gene	RNAi	Stock center	Construct ID	Construct target sequence	Migration defect (Primary screen)	Migration defect (Secondary screen): Mean ± [SD]
***alpha-catenin (α-cat)***	**20123^#^**	**VDRC**	**GD8808**	Same construct^*^	**89%**	**76% ± 0.07^#^**
	**40882**	**VDRC**	**GD8808**	Same construct^*^	**73%**	ND
	**107298**	**VDRC**	**KK107916**	**Independent construct**	**86%**	**66% ± 0.05**
*CAP*	106309	VDRC	KK107936	Independent construct	0.80%	2% ± 0.01
	19054	VDRC	GD8545	Independent construct	7%	4% ± 0.01
	30506	BL	HMS05250	Independent construct	11%	4% ± 0.03
	36663	BL	HMS01551	Independent construct	6.30%	5% ± 0.01
*Caskin*	24526	VDRC	GD7723	Same construct^*^	11%	9% ± 0.02
	25222	VDRC	GD7723	Same construct^*^	10%	9% ± 0.00
*CG3770*	4064	VDRC	GD2223	Overlap with KK101078 and HMJ2304^¥^	8%	9% ± 0.01^§^
	103556	VDRC	KK101078	Overlap with GD2223 and HMJ2304 ^¥^	26%	2% ± 0.01
	61262	BL	HMJ2304	Overlap with KK101078 and GD2223^¥^	9%	8% ± 0.01
*CG45049*	102985	VDRC	KK112983	Independent construct	13%	4% ± 0.01
	102025	VDRC	KK110412	Overlap with GD3956 and GD8606 ^¥^	8%	8% ± 0.01
	32403	VDRC	GD8606	Overlap with GD3956 and KK112983 ^¥^	20%	12% ± 0.02
	9673	VDRC	GD3956	Overlap with GD8606 and KK112983 ^¥^	8%	8%^§^
***Dachsous (ds)***	**36219**	**VDRC**	**GD14350**	**Independent construct**	**5%**	**14 ± 0.02**
	**4313**	**VDRC**	**GD2646**	**Independent construct**	**11%**	**12% ± 0.07**
	**32964**	**BL**	**HMS00759**	**Independent construct**	**ND**	**13% ± 0.05**
***Dreadlocks (dock)***	**37524**	**VDRC**	**GD4034**	**Independent construct**	**9%**	**19% ± 0.03**
	**37525**	**VDRC**	**GD4035**	**Unknown** ^†^	**11%**	**NA^§^**
	**107064**	**VDRC**	**KK102500**	**Independent construct**	**5%**	**4% ± 0.04**
	**27728**	**BL**	**JF02810**	**Independent construct**	**8%**	**13% ± 0.02**
***Fat***	**108863**	**VDRC**	**KK101190**	**Independent construct**	**11%**	**11% ± 0.04**
	**9396**	**VDRC**	**GD881**	**Independent construct**	**8%**	**11% ± 0.02**
*G protein alpha i subunit*	40890	BL	HMS02138	Overlap with JF0168^¥^ and HMS1273^¥^	20%	2% ± 0.02
	31133	BL	JF01608	Overlap with HMS02138^¥^ and HMS1273 ^¥^	12%	3% ± 0.02
	28150	VDRC	GD12576	Overlap with JF0168^¥^	5%	5% ± 0.01
	34924	BL	HMS01273	Overlap with JF0168 ^¥^ and HMS02138^¥^	16%	2% ± 0.01
*G protein alpha o subunit*	34653	BL	HMS01129	Independent construct	4%	3% ± 0.04
	110552	VDRC	KK109018	Overlap with GD8640^¥^	21%	3% ± 0.01
	19124	VDRC	GD8640	Overlap with KK109018^¥^	6%	15% ± 0.06
*Gliotactin*	37115	VDRC	GD1735	Same construct^*^	9%	10% ± 0.01
	37116	VDRC	GD1735	Same construct^*^	12%	6% ± 0.02
	107258	VDRC	KK105971	Independent construct	8%	2% ± 0.03
	38284	BL	HMS01737	Overlap with GD1735 ^¥^	10%	1% ± 0.01
	58115	BL	HMJ22052	Independent construct	10%	3% ± 0.04
***Lachesin (Lac)***	**35524**	**VDRC**	**GD12649**	**Overlap with KK107469 and HM05151^¥^**	**15%**	**10% ± 0.02**
	**107450**	**VDRC**	**KK107469**	**Overlap with GD12649 and HM05151^¥^**	**17%**	**5% ± 0.03**
	**38536**	**BL**	**HMS01756**	**Independent construct**	**23%**	**5% ± 0.02**
	**28940**	**BL**	**HM05151**	**Overlap with KK107469 and GD12649^¥^**	**ND**	**10% ± 0.01**
*Liprin-alpha*	106588	VDRC	KK100116	Independent construct	6%	5% ± 0.05
	51707	VDRC	GD7232	Independent construct	14%	7% ± 0.01
	53868	BL	HMC03183	Independent construct	19%	5% ± 0.06
*Lowfat*	32145	VDRC	GD7934	Overlap with KK102118 and JF03183 ^¥^	5%	ND
	32146	VDRC	GD7934	Overlap with KK102118 and JF03183 ^¥^	3%	ND
	107630	VDRC	KK102118	Overlap with GD7934 and JF03183 ^¥^	9.4%	ND
	28755	BL	JF03183	Overlap with KK102118 and GD7934^¥^	3.5%	ND
*Mesh*	40940	VDRC	GD3139	Independent construct	16%	3% ± 0.04
	6867	VDRC	GD3140	Unknown^†^	6%	NA
*Parvin*	11670	VDRC	GD3687	Overlap with KK102567^¥^	7.40%	8% ± 0.01
	105356	VDRC	KK102567	Overlap with GD3687^¥^	5%	2% ± 0.04
	42831	BL	HMS02523	Independent construct	19%	3% ± 0.02
***Roughest (rst)***	**27223**	**VDRC**	**GD14475**	Same construct^*^	**22%**	**16% ± 0.03**
	**27225**	**VDRC**	**GD14475**	Same construct^*^	**9.6%**	**11% ± 0.01**
	**951**	**VDRC**	**GD86**	**Overlap with GD14475^¥^**	**5%**	**4% ± 0.04**
	**28672**	**BL**	**JF03087**	**Independent construct**	**ND**	**10% ± 0.01**
*Schizo*						
	36625	VDRC	GD14895	Same construct^*^	7%	13% ± 0.03
	36627	VDRC	GD14895	Same construct^*^	1.50%	NA
	106168	VDRC	KK103616	Independent construct	14%	4% ± 0.03
	39060	BL	HMS01980	Overlap with GD14895^¥^	5%	3% ± 0.01
*Shroom*						
	47147	VDRC	GD16363	Independent construct	6%	5% ± 0.005
	100672	VDRC	KK106863	Independent construct	34%	7% ± 0.04
	107966	VDRC	KK108450	Overlap with HMS02190^¥^	9.7%	7% ± 0.02
	40942	BL	HMS02190	Overlap with KK108450^¥^	9.7%	7% ± 0.02
***Symplekin (Sym)***	**33469**	**VDRC**	**GD9722**	Same construct^*^	**14%**	**23% ± 0.1**
	**33470**	**VDRC**	**GD9722**	Same construct^*^	**23%**	**32% ± 0.02**
	**39041**	**BL**	**HMS01961**	Independent construct	**8%**	**6% ± 0.01**
*Vulcan*	46229	VDRC	GD16319	Same construct^*^	14%	3% ± 0.05
	46230	VDRC	GD16319	Same construct^*^	10%	6% ± 0.01
	40925	BL	HMS02173	Independent construct	4%	10% ± 0.03
***Wnt4***	**38011**	**VDRC**	**GD5347**	Same construct^*^	**23%**	**24%**
	**38010**	**VDRC**	**GD5347**	Same construct^*^	**7%**	**12% ± 0.02**
	**104671**	**VDRC**	**KK102348**	Independent construct	**11%**	**13% ± 0.06**
	**29442**	**BL**	**JF03378**	Overlap with GD5347^¥^	**10%**	**9% ± 0.01**
*Wunen*	51090	VDRC	GD15706	Same construct^*^	5.1%	ND
	51091	VDRC	GD15706	Same construct^*^	7%	ND
	6446	VDRC	GD1640	Overlap with GD15706^¥^	7.6%	ND
*mCherry*	35785	BL	VALIUM20-mCherry		2-11%	3% ± 0.02

Positive hits from the border cell RNAi screen are in bold text; ND, not determined; NA, stock not available to retest; SD, standard deviation; ¥, Overlapping target sequences either partial or identical; *, Same construct but independent insertions; §, RNAi line tested in two trials (stock dead or no longer available at the stock center); #, data from Chen et al., 2020; †, Stock no longer available and the targeted sequence is unknown.

### Immunostaining and imaging

Ovaries were dissected in Schneider’s *Drosophila* Medium (Thermo Fisher Scientific, Waltham, MA, USA). After dissection, ovaries were fixed in 4% formaldehyde (Polysciences, Inc., Warrington, PA, USA) in 0.1 M potassium phosphate buffer, pH 7.4 for 10 min. NP40 block (50 mM Tris-HCl, pH 7.4, 150 mM NaCl, 0.5% NP40, 5 mg/ml bovine serum albumin) was used for intermediate washes and antibody dilutions. Primary antibodies were obtained from Developmental Studies Hybridoma Bank (DSHB, University of Iowa, Iowa City, IA, USA) and used at the following dilutions: rat monoclonal anti-E-Cadherin 1:10 (DCAD2), mouse monoclonal anti-Armadillo 1:100 (N27A1), and mouse monoclonal anti-Singed 1:25 (Sn7C). Anti-rat or isotype-specific anti-mouse secondary antibodies conjugated to Alexa Fluor-488 or -568 (Thermo Fisher Scientific) were used at 1:400 dilution. 4’,6-Diamidino-2-phenylindole (DAPI, Millipore Sigma) was used at 2.5 µg/ml to label nuclei. Aqua-Poly/Mount (Polysciences, Inc.) was used to mount egg chambers on slides, a coverslip was added, and the mounting media allowed to harden for 3 days prior to microscope imaging. The stained egg chambers were imaged either using an upright Zeiss AxioImager Z1 microscope with Apotome.2 optical sectioning or on a Zeiss LSM 880 confocal microscope (KSU College of Veterinary Medicine Confocal Core), using a 20x 0.75 numerical aperture (NA) objective. Images were processed in Zeiss ZEN 2 or FIJI software. Figures were prepared in Adobe Photoshop 2021 and line drawings were made in Adobe Illustrator 2021 or Affinity Design.

### Graphs and statistics

Graphs were prepared in GraphPad Prism 8 and GraphPad Prism 9 (GraphPad Software, San Diego, CA, USA). For the secondary screen and subsequent analyses, three trials were performed for each RNAi line (*n* ≥ 30 egg chambers scored in each trial). The cutoff value for a migration defect was calculated based on the background mean migration defect (3% ±0.02) in control egg chambers (*c306-*GAL4 tsGAL80/+; +/UAS-mCherry RNAi). To determine genuine “hits” from the screen, RNAi lines with ≥10% migration defects were scored as positive hits in the primary and secondary screens. *P*-values were calculated using an unpaired two-tailed *t*-test in Microsoft Excel. For GBM regional and grade-dependent gene expression analyses, differences between groups were determined using a one-way ANOVA. N’s and *P*-values for each trial are included in the figure legends and tables.

## Results and discussion

### Identification of conserved brain tumor-associated adhesion genes

Cell–cell adhesion is essential for cells to stay connected during cohesive collective migration (Friedl and Mayor 2017). Reduction (or loss) of adhesion genes, such as E-cadherin (*Drosophila shotgun* [*shg*]), disrupts the integrity of the cluster and blocks the migration of the border cell cluster to the oocyte (Figure  1, D and E) (Niewiadomska *et al.* 1999; Sarpal *et al.* 2012; Desai *et al.* 2013; Cai *et al.* 2014; Raza *et al.* 2019; Chen *et al.* 2020). Many adhesion genes are conserved from flies to humans and could contribute to both border cell migration and GBM invasion (Figure  1F). To identify these conserved adhesion genes, we first performed a search of the *Drosophila* genome (FB2014_05), using GO CV terms associated with cell adhesion (see *Materials* *and Methods* for details; Figure  1G). It is important to note that while these “adhesion-related” candidate genes were originally chosen due to their known or predicted roles in cell adhesion, many of these genes have additional cellular roles, including cell-ECM interactions, cell signaling, cell polarity, as well as other functions. From the 133 fly genes associated with one or more of these terms, we identified likely human orthologs by analyzing their DIOPT scores (Table  1; Hu *et al.* 2011). Using these human orthologs, we performed a PubMed search for those genes to determine if there was an already-known association with either glioma or GBM. This allowed us to focus on genes that may have a novel association with brain tumors. The remaining 44 genes were then analyzed in the Repository of Molecular Brain Neoplasia Data (REMBRANDT), a database for transcript expression levels that are associated with brain tumor patient survival (Gusev *et al.* 2018). Ten genes were not found in REMBRANDT. Of the remaining 34 human genes, expression of 18 genes (13 fly genes) were associated with better (“positive”) patient survival while expression of 16 genes (13 fly genes) were associated with worse (“negative”) patient survival (Table  1). Many fly genes have multiple human orthologs. A few of these, for example *α-cat*, *G protein alpha i subunit*, and *G protein alpha o subunit*, have multiple human orthologs each of whose expression is associated with different predicted glioma patient outcomes (Table  1). For comparison, we have included any current known roles for these genes in cell migration or glioma (Table  1; Supplementary File S1). The 23 unique fly genes were chosen for further follow-up to determine their role, if any, in border cell collective migration.

### RNAi screen in border cells identifies eight genes associated with GBM

**Figure 2:**
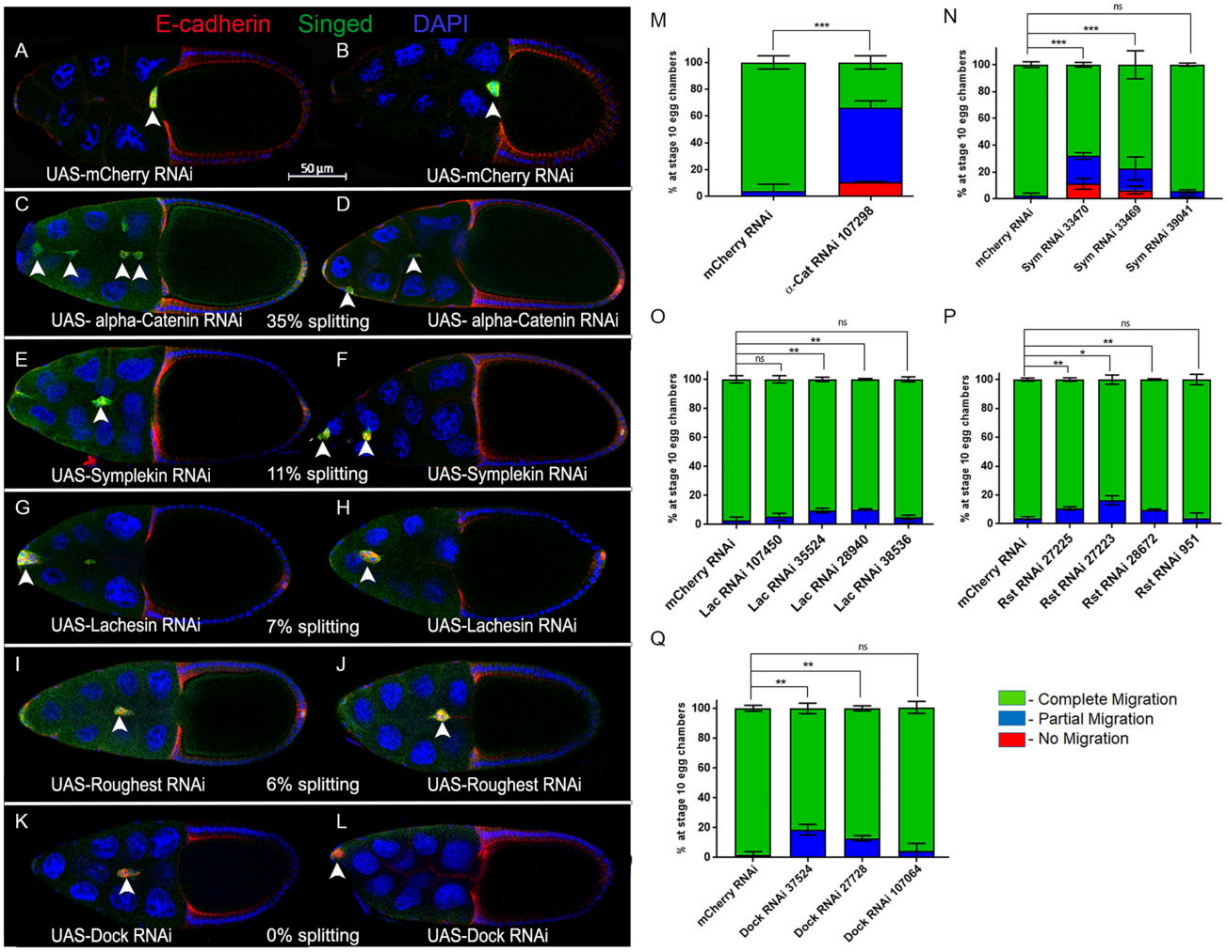
Cell adhesion and cell junction-associated genes whose RNAi knockdown impairs border cell migration. (A–L) Stage 10 egg chambers expressing RNAi for the indicated genes (or control) in border cells labeled for E-cadherin (red), a cell membrane and adhesion marker, Singed (green), which is highly expressed in and marks border cells, and DAPI to label all cell nuclei (blue). Two images are shown to indicate the general extent of phenotypes with RNAi knockdown for each gene. White arrowheads show the position of border cell clusters; the scale bar (A,B) indicates the image magnification for all images in the figure. Anterior is to the left. (A,B) Border cells expressing the control, *mCherry* RNAi, reach the oocyte at stage 10. (C–L) RNAi knockdown of *α-Catenin/α-Cat* (C and D, line v107298), *Symplekin/Sym* (E, line v33470; F, line v33469), *Lachesin/Lac* (G and H, line BL28940), *Roughest/Rst* (I and J, line v27223), and *Dock* (K, line v37524; L, line BL27728) driven by *c306*-GAL4 tsGAL80 disrupts the collective migration of border cells. The average percentage of egg chambers with border cell cluster splitting defects (% splitting) from the RNAi line with the strongest migration defect is indicated. (M–Q) Quantification of the extent of border cell migration (no migration, red; partial migration, blue; complete migration, green) in stage 10 egg chambers expressing the indicated RNAi lines for *α-Cat* (M), *Sym* (N), *Lac* (O), *Rst* (P), and *Dock* (Q) along with the matched control *mCherry* RNAi. Error bars represent SEM for three trials, *n* ≥ 30 egg chambers in each trial. *P < 0.05; **P < 0.005; ***P < 0.001, unpaired two-tailed *t*-test.

For the primary screen, multiple RNAi lines were used to specifically target and knock down each of the 23 conserved fly adhesion and adhesion-related genes in border cells (Table  2). These lines include independent targeted sequences, overlapping targeted sequences, and independent insertions of the same RNAi construct (see Table  2). Some RNAi lines used in this screen were validated in different *Drosophila* systems, whereas others have not yet been reported in published studies (FlyBase; Supplementary Table S1 and File S2). We drove expression of the respective UAS-RNAi lines using *c306*-GAL4 tsGAL80, a follicle cell driver highly enriched in border cells prior to and during their migration; tsGAL80 was used to bypass potential early lethality (Figure  1, A–C). All border cell clusters from control (*c306*-GAL4 tsGAL80/+; +/UAS-mCherry RNAi) egg chambers completed their migration by stage 10 (Figure  2, A and B; Table  2). Twenty-one of these genes displayed a migration defect above the minimum cutoff of ≥10% with at least one RNAi line (see *Materials* *and Methods*).

**Figure 3:**
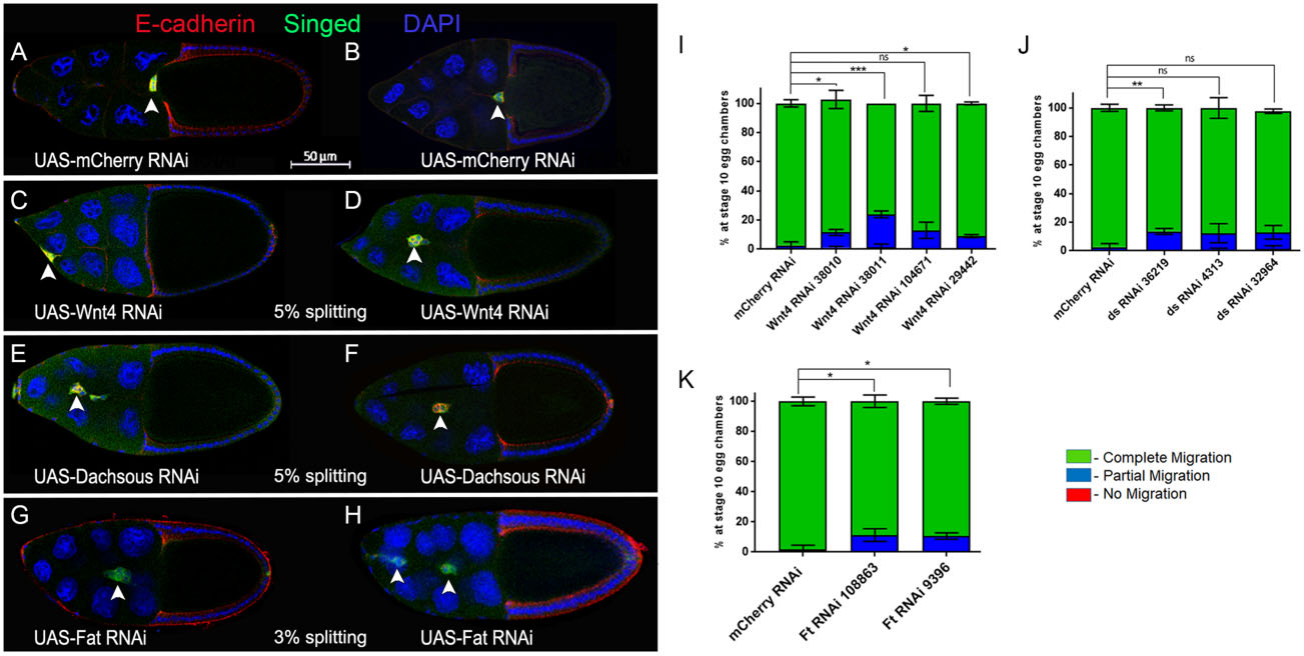
Atypical cadherins and planar cell polarity genes whose RNAi knockdown impairs border cell migration. (A–H) Stage 10 egg chambers expressing RNAi for the indicated genes (or control) in border cells labeled for E-cadherin (red), a cell membrane and adhesion marker, singed (green), which is highly expressed in border cells, and DAPI to label all cell nuclei (blue). Two images are shown to indicate the general extent of phenotypes with RNAi knockdown for each gene. White arrowheads show the position of border cell clusters; the scale bar (A,B) indicates the image magnification for all images in the figure. Anterior is to the left. (A,B) Border cells expressing the control, *mCherry* RNAi, reach the oocyte at stage 10. (C–H) RNAi knockdown of *Wnt4* (C and D, line v38011), *Dachsous/ds* (E, line 32964; F, line v4313), and *Fat/ft* (G and H, line BL28940) driven by *c306*-GAL4 tsGAL80 disrupts the collective migration of border cells. The average percentage of egg chambers with border cell cluster splitting defects from the RNAi line with the strongest migration defect is indicated. (I–K) Quantification of border cell migration (no migration, red; partial migration, blue; complete migration, green) in stage 10 egg chambers expressing the indicated RNAi lines for *Wnt4* (I), *ds* (J), and *ft* (K) along with the matched control *mCherry* RNAi. Error bars represent SEM for three trials, *n* ≥ 30 egg chambers in each trial. *P < 0.05; **P < 0.005; ***P < 0.001, unpaired two-tailed *t*-test.

To further determine which of these genes were genuine hits, we retested the RNAi lines in a secondary screen. Each RNAi line was crossed to *c306*-GAL4 tsGAL80 three times and scored for the ability of border cells to complete their migration to the oocyte. For three genes (*ds, Lac*, and *rst*), additional RNAi lines were obtained from stock centers and tested. We specifically analyzed if RNAi border cells failed to initiate migration (“no migration”), stopped along the migration pathway but did not reach the oocyte (“partial migration”), reached the oocyte (“complete migration”), or if clusters had defective cohesion and split into multiple parts (“% splitting”). Control border cells completed their migration to the oocyte by stage 10 (Figures  2, A and B and 3, A and B; Table  2). We found that knockdown of eight genes, *α-Cat, Sym*, *Lac, rst*, *dock*, *Wnt4*, *ds*, and *ft*, consistently disrupted border cell migration with at least two RNAi lines, providing more confidence that these genes are required for collective cell migration (Figures  2 and 3; Table  2). Border cell migration defects upon knockdown of these genes ranged from 10% to 76% depending on the gene and the RNAi line; some RNAi lines for these genes had less than 10% migration defects. Below we report and discuss the results for these eight genes in more detail.

*Adherens junction genes*: α-Cat (human CTNNA1, CTNNA2, and CTNNA3) is a critical component of the cadherin-catenin complex that regulates adherens junctions by linking E-cadherin and β-catenin to the F-actin cytoskeleton (Maiden and Hardin 2011). E-cadherin is required for adhesion of border cells to the nurse cell substrate, which provides traction for border cells to keep moving forward and thus facilitates forward movement while maintaining tension-based directional motility (Niewiadomska *et al.* 1999; Cai *et al.* 2014). α-Cat was the strongest candidate from our primary screen (Table  2), and we recently described the phenotypes for *α-Cat* knockdown in detail (Chen *et al.* 2020). *α-Cat* was knocked down using two independent RNAi lines, which reduced α-Cat protein levels in border cells (Chen *et al.* 2020). *α-Cat* RNAi strongly disrupted migration, with 66–76% border cells failing to complete their migration (Figure  2, C, D, and M; Table  2). Border cell clusters deficient for *α-Cat* also had significant cohesion defects, with the cluster splitting into two or more parts in 35% of egg chambers (Figure  2, C and D). Thus, *Drosophila α-Cat* is required for both successful border cell migration and for proper cohesion of cells within the cluster (this study; Sarpal *et al.* 2012; Desai *et al.* 2013; Chen *et al.* 2020). The role for α-Cat in cluster cohesion and migration closely resembles that of β-Cat (*Drosophila* Armadillo) and E-cadherin, thus it is likely that α-Cat functions in the classical cadherin-catenin complex in border cells (Niewiadomska *et al.* 1999; Sarpal *et al.* 2012; Desai *et al.* 2013; Cai *et al.* 2014; Chen *et al.* 2020).

*Other junctional genes:* Four genes, *Sym*, *Lac*, *rst*, and *dock*, encode proteins that localize to various types of cell junctions and/or are known to regulate cell adhesions. Sym (human SYMPK) is a scaffolding protein, which along with other polyadenylation factors, forms a complex that mediates processing of polyadenylated and histone mRNAs but also functions at tight junctions (Keon *et al.* 1996; McCrea *et al.* 2009; Sullivan *et al.* 2009). During *Drosophila* oogenesis, Sym is required for histone pre-mRNA processing in the histone locus body during endoreplication of the follicular epithelium (Tatomer *et al.* 2014). Later in oogenesis, Sym protein localizes to the tricellular junctions of follicle cells. Here, Sym may facilitate cytoplasmic mRNA polyadenylation and thus translation of mRNAs required to regulate and/or maintain adhesion at cell junctions (Tatomer *et al.* 2014). Border cells expressing *Sym* RNAi had significant migration defects along with splitting of the cluster (Figure  2, E, F, and N; Table  2). The two strongest *Sym* RNAi lines (VDRC 33469 and 33470), which target the same region of the *Sym* gene, caused significant migration defects, with 5–10% of border cells failing to start migration and an additional 18–22% failing to reach the oocyte. *Sym* RNAi border cell clusters had cohesion defects, with 11% of clusters visibly splitting apart. A third independent RNAi line (BL 39041) did not impair migration (Figure  2N). Based on our observed phenotypes and the known roles for Sym, we speculate that *Sym* may maintain cell–cell contacts between border cells during collective migration, possibly through regulation of as-yet-unknown targets by mRNA polyadenylation at cell–cell junctions.

Lac (human LSAMP and NEGR1) is a membrane-localized protein with three extracellular immunoglobulin-like (Ig-like) domains that can mediate cell–cell adhesion (Finegan and Bergstralh 2020). Lac localizes to both immature and mature basolateral septate junctions and is required for tracheal morphogenesis in *Drosophila* (Llimargas *et al.* 2004). Knockdown of *Lac* by four RNAi lines, which together target two nonoverlapping regions of the *Lac* gene, mildly disrupted migration and cohesion of the cluster (Figure  2, G, H, and O; Table  2). Two *Lac* RNAi lines (VDRC 35524 and BL 28940) disrupted migration in 11% of egg chambers, whereas two RNAi lines (VDRC 107450 and BL 38536) had fewer migration defects and were not significantly different from control (Figure  2O; Table  2). While the phenotypes caused by *Lac* RNAi knockdown are mild, recent work by Alhadyian *et al.* found that four additional septate junction proteins, Macroglobulin complement-related (Mcr), Contactin, Neurexin-IV and Coracle, localize to border cells and are required for both border cell cluster migration and cohesion (Alhadyian *et al.* 2021). Because border cells do not have mature septate junctions (which form the tight occluding junctions), septate junction proteins may instead regulate cluster polarity and/or adhesion during migration (Alhadyian *et al.* 2021). Thus, Lac is likely to have a specific role in border cell migration along with other septate junction proteins. Further work will be needed to determine if the mild phenotypes observed with *Lac* RNAi are due to partial knockdown or to redundancy with other septate junction genes.

Rst (human KIRREL1, KIRREL2, and KIRREL3) is a member of the Irre Cell Recognition Module (IRM) family of transmembrane proteins. In particular, Rst encodes an immunoglobulin superfamily cell adhesion molecule (IgCAM) with five Ig-like domains (Finegan and Bergstralh 2020). IRM proteins, including Rst, control the adhesion and patterning of various tissues including the developing ommatidia in the *Drosophila* eye (Bao and Cagan 2005; Johnson *et al.* 2011; Finegan and Bergstralh 2020). Border cells expressing *rst* RNAi showed consistent though mild migration defects with three RNAi lines (VDRC 27223, VDRC 27225, and BL 28672), which in total target two nonoverlapping regions of the *rst* gene. Migration defects ranged from 10% to 16% (Figure  2, I, J, and P; Table  2). Cluster cohesion was mildly affected (6% of clusters split apart; Figure  2I). A fourth RNAi line did not disrupt migration or cohesion compared to control (Figure  2P; VDRC 951). Interestingly, Rst is required for progression through *Drosophila* adult oogenesis, including development of the germline (Valer *et al.* 2018; Ben-Zvi and Volk 2019). Rst is also expressed in follicle cells prior to the stages that border cells develop from the follicle cell epithelium (Valer *et al.* 2018), further supporting a later role in border cell migration.

Dock (human NCK1) is an SH2/SH3 domain-containing adaptor protein involved in receptor tyrosine kinase signaling, actin regulation, cell adhesion, and other processes (Buday *et al.* 2002; Chaki and Rivera 2013). In *Drosophila*, Dock regulates axon guidance, myoblast fusion during embryonic development, and ring canal morphogenesis in the ovarian germline-derived nurse cells (Garrity *et al.* 1996; Rao and Zipursky 1998; Kaipa *et al.* 2013; Stark *et al.* 2021). Knockdown of *dock* in border cells, using two independent RNAi lines that target nonoverlapping regions of the *dock* gene (VDRC 37524 and BL 27728), resulted in migration defects but did not disrupt cohesion of border cells (Figure  2, K, L, and Q; Table  2). Specifically, *dock* RNAi disrupted migration in 13–19% of stage 10 egg chambers (Figure  2Q; Table  2). One RNAi line (VDRC 107064) did not impair border cell migration but showed mild splitting (6%), whereas another line (VDRC 37525) from the primary screen was no longer available so could not be confirmed in the secondary screen (Figure  2Q; Table  2). Dock is required for myoblast fusion during muscle formation by regulating cell adhesion and F-actin (Kaipa *et al.* 2013). In this context, Dock colocalizes with and/or binds to several cell adhesion proteins from the IgCAM superfamily including Rst, one of the genes identified in this screen (see above). In addition, Dock genetically and biochemically interacts with the Ste20-like serine-threonine kinase Misshapen (Msn) to control motility of photoreceptor growth cones in the developing eye (Ruan *et al.* 1999). Notably, Msn is required for border cell migration, where it is required for the formation of polarized protrusions and coordinated actomyosin contractility of the cluster (Plutoni *et al.* 2019). Thus, it will be of interest in the future to determine if Dock, Rst, and Msn interact to control border cell migration.

*Atypical cadherins and planar cell polarity genes:* Three genes, *Wnt4*, *ds*, and *ft* encode proteins with annotated roles in both planar cell polarity and cell–cell adhesion (FlyBase; Figure  3; Table  2). Wnt4 (human WNT9A) is a conserved secreted protein of the Wnt family, which regulates cell adhesion through recruitment of focal adhesion complexes during the migration of epithelial cells in the pupal ovary (Cohen *et al.* 2002). We tested four RNAi lines for *Wnt4*, which in total target two independent regions of the gene. Migration defects for the four tested *Wnt4* RNAi lines ranged from 9% to 23% (Figure  3, C, D, and I; Table  2). These data suggest a role for Wnt4 in regulating border cell movement. Previous studies suggested that Wnt4 participates in establishing planar polarity within the developing eye and wing (Lim *et al.* 2005; Wu *et al.* 2013). Indeed, several core planar cell polarity genes including *frizzled* and *disheveled* regulate border cell migration (Bastock and Strutt 2007). However, recent studies that used multiple gene knockouts now indicate that the Wnt family of proteins, including Wnt4, are not required for *Drosophila* planar cell polarity (Ewen-Campen *et al.* 2020; Yu *et al.* 2020). Thus, we favor a role for Wnt4 in the movement and adhesion of border cells, similar to what was found during earlier stages of *Drosophila* ovarian development (Cohen *et al.* 2002).

Ds (human DCHS1) and Ft (human FAT4) encode large protocadherin proteins, each of which has multiple extracellular cadherin repeats (27 for Ds and 34 for Ft) (Fulford and McNeill 2020). Heterophilic binding between Ds and Ft via their extracellular domains is essential for cell–cell communication, particularly in the regulation of tissue growth through Hippo signaling and planar polarization of various tissues (Matakatsu and Blair 2004; Bosveld *et al.* 2016; Blair and McNeill 2018; Fulford and McNeill 2020). Knockdown of *ds* with any of three independent RNAi lines (VDRC 36219, VDRC 4313, and BL 32964) mildly disrupted migration, ranging from 12% to 14% of border cells failing to reach the oocyte (Figure  3, E, F, and J; Table  2). *ds* RNAi border cell clusters only displayed mild cohesion defects, with 5% of clusters splitting apart (Figure  3E). Two independent RNAi lines that target *ft* (VDRC 108863 and VDRC 9396) also showed consistent though mild migration defects (11–13%), with only a few clusters (3%) splitting apart (Figure  3, G, H, and K; Table  2). Interestingly, *ds* is required for the collective directional migration of *Drosophila* larval epidermal cells (LECs) during morphogenesis of the pupal abdominal epithelium (Bischoff 2012; Arata *et al.* 2017). An imbalance in Ds protein levels between LECs during collective migration is detected by Ft at cell junctions leading to the formation of lamellipodia at the posterior side of the LECs (Arata *et al.* 2017). Further experiments will be needed to determine if Ft and Ds similarly coordinate protrusions in border cells or regulate some other aspect of border cell collective migration.

The RNAi screen approach used in this study allows rapid functional testing of genes but comes with technical limitations (Perrimon *et al.* 2010; Booker *et al.* 2011). Possible caveats of RNAi-mediated knockdown include potential off-target effects (“false positives”), RNAi constructs that fail to knock down a given gene’s function (“false negatives”), genomic-insertion effects that reduce expression of an RNAi transgene and thus knockdown efficiency, transient or partial functional knockdown in cells and tissues by a given RNAi transgene, and/or compensation by related genes. We attempted to address some of these potential RNAi issues. To control for general activation of the RNAi machinery, we performed RNAi knockdown to monomeric Cherry (mCherry), a fluorescent protein not normally found in *Drosophila* (*e.g.*, Figure  2, A, B, and M; Table  2). Whenever possible, to provide better confidence of RNAi-mediated knockdown results, we tested multiple RNAi lines for each gene, which include RNAi transgenes that target independent gene regions and independent insertions that target overlapping gene sequences (Table  2). Many of these RNAi lines have been used in other *Drosophila* screens and other functional studies, with various phenotypes observed such as pupal lethality, bristle defects, and others (FlyBase; Supplementary Table S1 and Supplementary File S2).

Partial functional knockdown could also be due to expression levels of the GAL4-UAS system itself. We included tsGAL80 in our genetic crosses to prevent early GAL4-UAS-RNAi expression and potential lethality prior to the stages of oogenesis when border cells migrate. Under the experimental conditions of the screen (see *Materials* *and Methods*), it is possible that leaky tsGAL80 could further dampen expression of GAL4-UAS-RNAi in border cells. However, we have previously used the same GAL4 line, *c306-*GAL4, in combination with tsGAL80 under similar experimental conditions to drive RNAi-mediated knockdown in border cells; RNAi for at least two genes reduced levels of the respective proteins within border cells (Aranjuez *et al.* 2012, 2016). As with all RNAi screens, further follow-up experiments with loss-of-function mutant alleles or cell-specific CRISPR-Cas9 are needed to confirm the specificity of the phenotypes (Mohr *et al.* 2014). Future experiments include performing live cell imaging and other cellular assays to determine when each of these genes is required and how the genes precisely regulate collective border cell migration.

### Analysis of regional expression of border cell screen hits in GBM tumors

**Figure 4:**
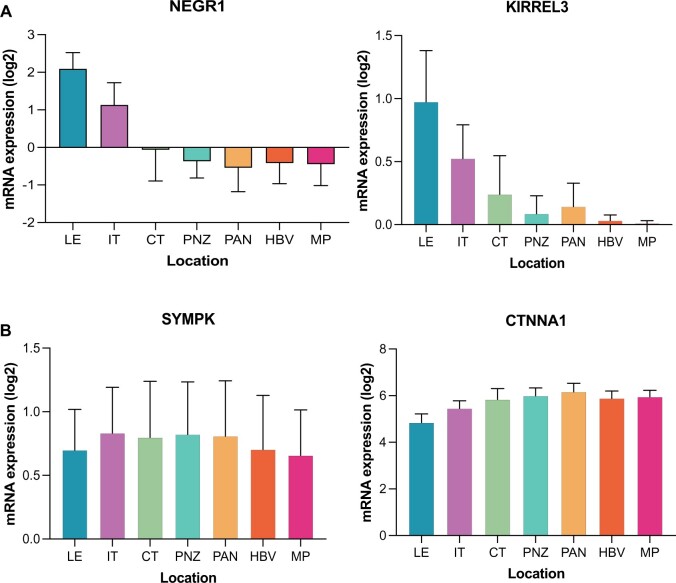
Regional expression of representative human ortholog adhesion-related genes in GBM patient tumors. (A) Expression of human orthologs of neuronal growth regulator 1 (NEGR1) and kirre like nephrin family adhesion molecule 3 (KIRREL3) is significantly enriched in the LE and IT compared to other tumor regions, including the CT, PNZ, PAN, HBV, and MP. (B) In contrast, expression of human orthologs symplekin (SYMPK) and catenin alpha 1 (CTNNA1) demonstrated little to no significant change when comparing different regions of tumor. Data from the Ivy GAP are shown as mean expression ± SD across GBM tumor regions. Statistics are shown in Supplementary Table S2: *P < 0.05; **P < 0.01; ***P < 0.001, one way ANOVA with Tukey HSD.

Based on the results of the functional *Drosophila* screen, we next sought to link individual genes to invasion in human GBM patient tumors. We first assessed the Ivy GAP database that provides regional RNA expression across anatomically defined regions of tumors ranging from the tumor core to the infiltrating edge (see *Materials* *and Methods*). Using this database, we found that NEGR1 and KIRREL3 were specifically enriched in anatomical regions with elevated invasion potential, namely the leading edge (LE) and infiltrating tumor (IT), compared to all other assessed anatomical regions (Figure  4A; Supplementary Table S2). These regions included cellular tumor (CT), perinecrotic zone (PNZ), pseudopalisading cells around necrosis (PAN), hyperplastic blood vessels (HBV), and microvascular proliferation (MP). In addition, CTNNA2 had significant expression in the LE and IT regions though was also expressed in other regions of the tumor (Supplementary Figure S1 and Table S2). However, we also observed some *Drosophila* screen hits that did not demonstrate regional heterogeneity in terms of expression, such as SYMPK and CTNNA1 (Figure  4B; Supplementary Table S2). Other genes had a mixture of expression profiles across human GBM anatomical regions (CTNNA3, DCHS1, FAT4, and KIRREL1, KIRREL2, NCK1; Supplementary Figure S1 and Table S2). WNT9A was not found in the Ivy GAP database. It is worth noting that this initial validation approach takes advantage of regional differences within the same GBM tumor. Therefore, such GBM anatomical expression surveys may be a better surrogate of cellular invasion than expression in GBM compared to lower grade or nonneoplastic neural tissue; these latter analyses rely on gene expression in tissue obtained mainly from the core of the tumor and may miss areas of the tumor that undergo active invasion (Supplementary Figures S2 and S3). Nonetheless, we observed a variety of human adhesion ortholog gene-dependent increases or decreases in GBM tumors compared to lower-grade or nonneoplastic neural tissue (Supplementary Figures S2 and S3). Together, these assessments provide a first step in validating novel, conserved molecular mechanisms of GBM invasion for future therapeutic development. Invasive GBM is thought to be driven by CSCs, which can migrate and invade as single cells, finger-like collectives, or as a mixture of migration modes (Cheng *et al.* 2011; Volovetz *et al.* 2020). Human Rap1a, originally identified in a *Drosophila* screen of collective border cell migration, influences CSC-mediated GBM cell invasion (Aranjuez *et al.* 2012; Volovetz *et al.* 2020). Interestingly, knocking down *Sym* and *α-Cat* in the border cells caused the most severe migration and cluster cohesion defects. While the respective human orthologs SYMPK, CTNNA1, and CTNNA2 did not show regional tumor heterogeneity, they are each expressed in GBM tumors and/or are generally elevated in different grades of glioma including GBM (Grade IV; Supplementary Figure S2).

A limitation of this study involves the use of GBM expression and survival data from TCGA. The samples used to curate this database are primarily taken from core biopsies of resected GBM tumors, which restricts the availability of invasive cells and therefore the use of these data for assessing invasive potential. Similarly, direct associations between expression and survival may be impacted by variable gene expression across different regions of the tumor. To counteract this, we used the Ivy GAP database to provide additional information about expression in each tumor region. Conversely, a large proportion of cells in the LE and IT of the Ivy atlas are nontumoral, which may confound interpretation of regional expression. It should also be noted, however, that expression alone is not necessarily indicative of function and that these studies are being used as a foundation upon which to build future studies.

## Conclusion

GBM, the most common primary malignant brain tumor in adults, is also one of the most lethal (Ostrom *et al.* 2014, 2018). These tumors are highly invasive and possess a self-renewing CSC population. CSCs are highly invasive and can migrate either individually or collectively (Cheng *et al.* 2011; Volovetz *et al.* 2020). Here, we used a human GBM-informed approach to identify conserved regulators of adhesion during collective cell migration and invasion, particularly focused on testing genes in the border cell model. We identified eight adhesion-related *Drosophila* genes (orthologs of 13 human genes) associated with glioma patient survival. Of the eight adhesion-related *Drosophila* genes found to be essential for collective cell migration, two human orthologs, NEGR1 and KIRREL3 showed significant regional enrichment in the LE and IT of human GBM tumors, areas associated with enhanced cell invasion. CTNNA2 was expressed in these invasive regions, though was also expressed at high levels in other regions of the tumor. Knockdown of these eight genes disrupted border cell migration to varying degrees, with two genes *α-cat* and *Sym* significantly disrupting both cohesion of the cluster and successful cell migration. Although the objective of this study was broadly directed toward understanding the adhesion-associated roles of genes in collective cell migration and invasion, many of these genes may have additional functions apart from cell adhesion. These eight *Drosophila* genes thus represent a starting point to further investigate the specific mechanisms by which these genes regulate normal collective cell migration. Future experiments using loss-of-function alleles and live imaging approaches are required to confirm the adhesion-related, or other, functions of these genes in the border cell system. In addition, whether these genes function through an adhesion-dependent or -independent manner in GBM tumors needs to be determined with follow-up experiments, using both mammalian and nonmammalian models of GBM, including *Drosophila* glioma models (Agnihotri *et al.* 2016; Chen *et al.* 2018; Chen and Read 2019; Chi *et al.* 2019; Gangwani *et al.* 2020; Shahzad *et al.* 2021). Overall, the strategy used in this study has the potential to identify new genes and conserved mechanisms that drive collective cell migration of normal cells and those in invasive cancers such as GBM.

## Data availability

Strains are available upon request. The authors affirm that all data necessary for confirming the conclusions of the article are present within the article, figures, and tables. Table  2 contains the complete results of the screen, including the RNAi lines tested, availability from the public stock centers (BDSC “BL” and VDRC), and detailed results from the primary and secondary screens. Supplementary Table S2 includes statistics for Figure  4 and Supplementary Figure S1. Supplementary Figure S1 shows the regional expression of the rest of the human orthologs in GBM patient tumors. Supplementary Figure S2 shows the expression of human ortholog adhesion genes in different glioma tumor grades. Supplementary Figure S3 shows a comparison of human ortholog adhesion gene expression in GBM *vs* nontumor brain tissue.

Supplementary material is available at *G3* online.

## Supplementary Material

jkab356_Supplementary_Figure1

jkab356_Supplementary_Figure2

jkab356_Supplementary_Figure3

jkab356_Supplementary_Figure4

jkab356_Supplementary_Figure5

jkab356_Supplementary_Table1

jkab356_Supplementary_Table2

jkab356_Supplementary_Figures-Tables-Captions
