# Supplementary figures and images for: A Drosophila RNAi screen reveals conserved glioblastoma-related adhesion genes that regulate collective cell migration
Source: G3 (Bethesda). 2021 Oct 11;12(1):jkab356. doi: 10.1093/g3journal/jkab356 (PMC8728034; doi:10.1093/g3journal/jkab356)

**CTNNA2**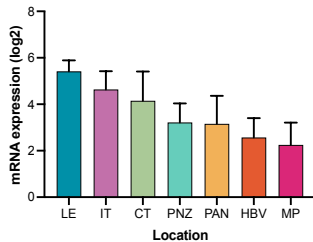**CTNNA3**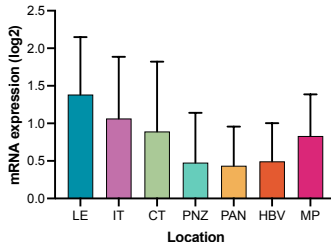**DCHS1**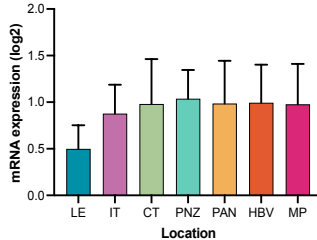**FAT4**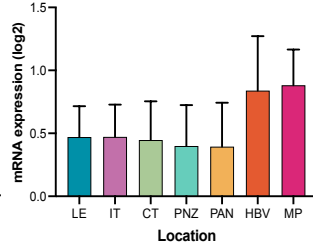**KIRREL1**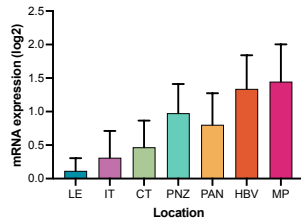**KIRREL2**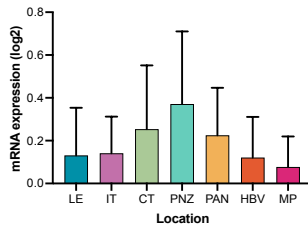**NCK1**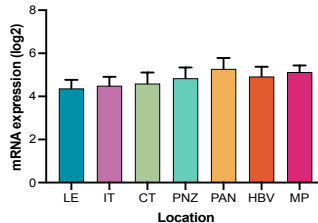

Supplement: jkab356_Supplementary_Figure1 [file jkab356_supplementary_figure1.pdf]

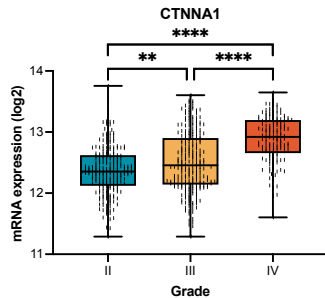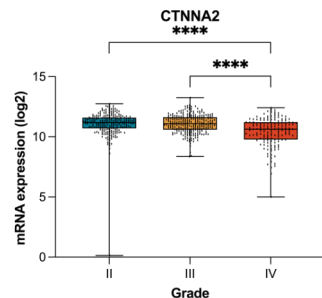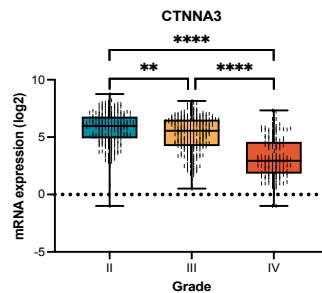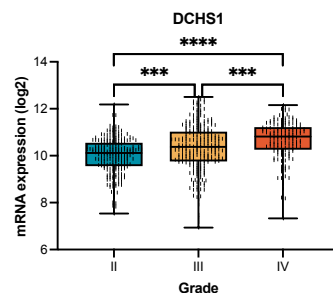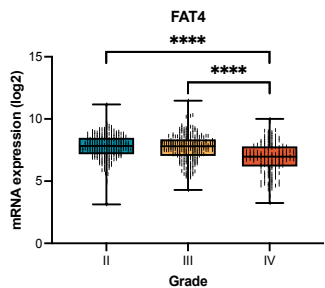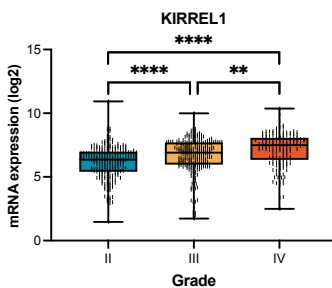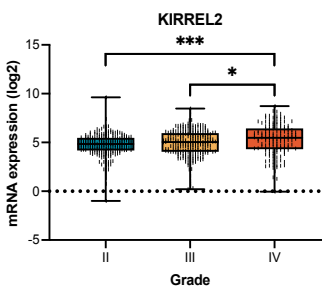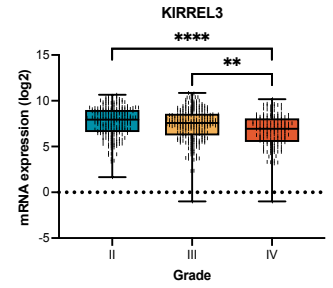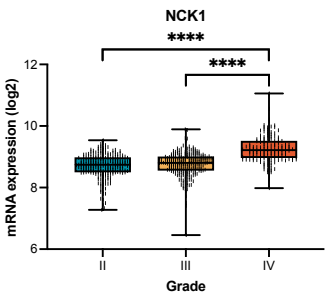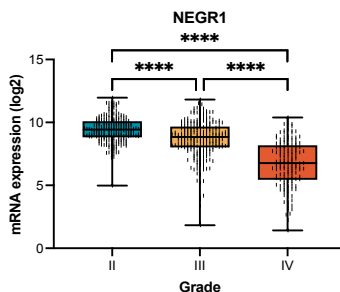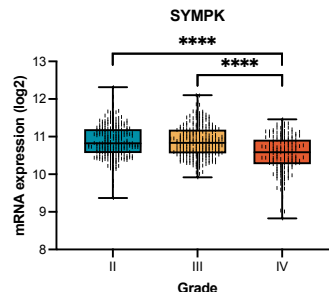

Supplement: jkab356_Supplementary_Figure2 [file jkab356_supplementary_figure2.pdf]

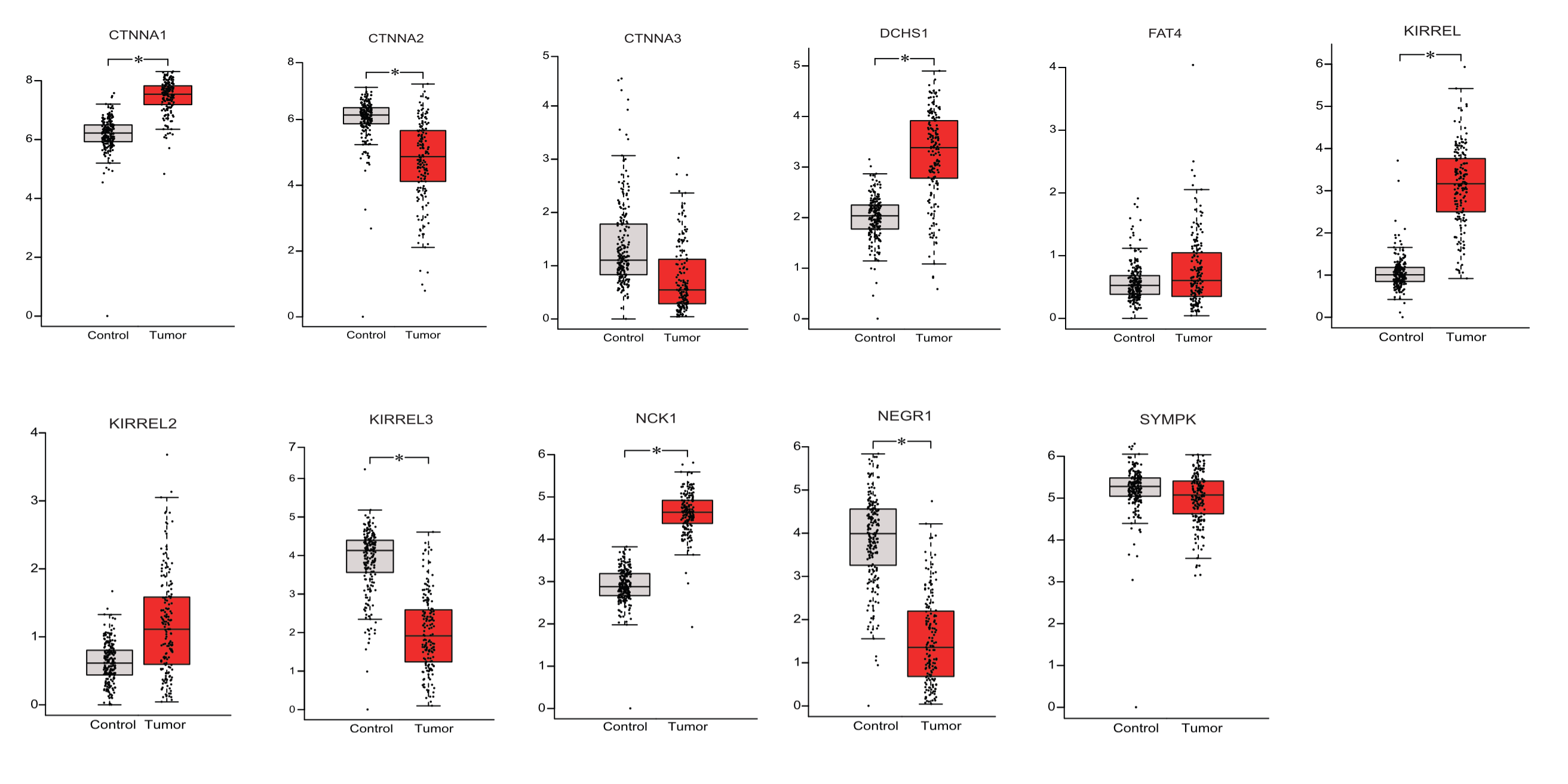

Supplement: jkab356_Supplementary_Figure3 [file jkab356_supplementary_figure3.zip › jkab356_Supplementary_Figure3.tif]
