# Supplementary material for: A Drosophila RNAi screen reveals conserved glioblastoma-related adhesion genes that regulate collective cell migration
Source: G3 (Bethesda). 2021 Oct 11;12(1):jkab356. doi: 10.1093/g3journal/jkab356 (PMC8728034; doi:10.1093/g3journal/jkab356)
Supplement: jkab356_Supplementary_Figure4 [file jkab356_supplementary_figure4.pdf]

Supplementary File 1. List of references for Table 1.

1. Benjamin, J. M., A. V. Kwiatkowski, C. Yang, F. Korobova, S. Pokutta *et al*., 2010 AlphaE-catenin regulates actin dynamics independently of cadherin-mediated cell-cell adhesion. J Cell Biol 189: 339–352.
2. Bunker, E. N., G. E. Wheeler, D. A. Chapnick, and X. Liu, 2021 Suppression of α-catenin and adherens junctions enhances epithelial cell proliferation and motility via TACE-mediated TGF-α autocrine/paracrine signaling. Mol Biol Cell 32: 348–361.
3. Matsuzawa, K., T. Himoto, Y. Mochizuki, and J. Ikenouchi, 2018 α-Catenin Controls the Anisotropy of Force Distribution at Cell-Cell Junctions during Collective Cell Migration. Cell Rep 23: 3447–3456.
4. Seddiki, R., G. H. N. S. Narayana, P.-O. Strale, H. E. Balcioglu, G. Peyret *et al*., 2018 Force-dependent binding of vinculin to α-catenin regulates cell-cell contact stability and collective cell behavior. Mol Biol Cell 29: 380–388.
5. Vassilev, V., A. Platek, S. Hiver, H. Enomoto, and M. Takeichi, 2017 Catenins Steer Cell Migration via Stabilization of Front-Rear Polarity. Developmental Cell 43: 463-479.e5.
6. Carvalho, J. R., I. C. Fortunato, C. G. Fonseca, A. Pezzarossa, P. Barbacena *et al*., 2019 Non-canonical Wnt signaling regulates junctional mechanocoupling during angiogenic collective cell migration. Elife 8: e45853.
7. Wood, M. N., N. Ishiyama, I. Singaram, C. M. Chung, A. S. Flozak *et al*., 2017 α-Catenin homodimers are recruited to phosphoinositide-activated membranes to promote adhesion. J Cell Biol 216: 3767–3783.
8. Schmid, M.-T., F. Weinandy, M. Wilsch-Bräuninger, W. B. Huttner, S. Cappello *et al*., 2014 The role of α-E-catenin in cerebral cortex development: radial glia specific effect on neuronal migration. Frontiers in Cellular Neuroscience 8: 215.
9. Cui, Y., and S. Yamada, 2013 N-Cadherin Dependent Collective Cell Invasion of Prostate Cancer Cells Is Regulated by the N-Terminus of α-Catenin. PLOS ONE 8: e55069.
10. Weber, G. F., M. A. Bjerke, and D. W. DeSimone, 2012 A Mechanoresponsive Cadherin-Keratin Complex Directs Polarized Protrusive Behavior and Collective Cell Migration. Developmental Cell 22: 104–115.
11. Jurado, J., J. de Navascués, and N. Gorfinkiel, 2016 α-Catenin stabilises Cadherin–Catenin complexes and modulates actomyosin dynamics to allow pulsatile apical contraction. Journal of Cell Science 129: 4496–4508.
12. Wang, Y.-C., Z. Khan, and E. F. Wieschaus, 2013 Distinct Rap1 Activity States Control the Extent of Epithelial Invagination via α-Catenin. Developmental Cell 25: 299–309.
13. Ji, H., J. Wang, B. Fang, X. Fang, and Z. Lu, 2011 α-Catenin inhibits glioma cell migration, invasion, and proliferation by suppression of β-catenin transactivation. J Neurooncol 103: 445–451.
14. Shinoura, N., N. E. Paradies, R. E. Warnick, H. Chen, J. J. Larson *et al*., 1995 Expression of N-cadherin and alpha-catenin in astrocytomas and glioblastomas. Br J Cancer 72: 627–633.
15. Dondi, C., B. Bertin, J.-P. Da Ponte, I. Wojtowicz, K. Jagla *et al*., 2021 A polarized nucleus-cytoskeleton-ECM connection in migrating cardioblasts controls heart tube formation in Drosophila. Development 148: dev192146.
16. Jammrath, J., I. Reim, and H. Saumweber, 2020 Cbl-Associated Protein CAP contributes to correct formation and robust function of the Drosophila heart tube. PLoS One 15: e0233719.
17. Ichikawa, T., M. Kita, T. S. Matsui, A. I. Nagasato, T. Araki *et al*., 2017 Vinexin family (SORBS) proteins play different roles in stiffness-sensing and contractile force generation. J Cell Sci 130: 3517–3531.

18. Arata, M., K. Sugimura, and T. Uemura, 2017 Difference in Dachsous Levels between Migrating Cells Coordinates the Direction of Collective Cell Migration. Dev Cell 42: 479-497.e10.
19. Dearborn, R., and S. Kunes, 2004 An axon scaffold induced by retinal axons directs glia to destinations in the Drosophila optic lobe. Development 131: 2291–2303.
20. Zakaria, S., Y. Mao, A. Kuta, C. F. de Sousa, G. O. Gaufo *et al.*, 2014 Regulation of neuronal migration by Dchs1-Fat4 planar cell polarity. Curr Biol 24: 1620–1627.
21. Mangione, F., and E. Martín-Blanco, 2018 The Dachsous/Fat/Four-Jointed Pathway Directs the Uniform Axial Orientation of Epithelial Cells in the Drosophila Abdomen. Cell Rep 25: 2836-2850.e4.
22. Fulford, A. D., and H. McNeill, 2020 Fat/Dachsous family cadherins in cell and tissue organisation. Curr Opin Cell Biol 62: 96–103.
23. Berg, C. A., 2005 The Drosophila shell game: patterning genes and morphological change. Trends Genet 21: 346–355.
24. Su, Y. C., C. Maurel-Zaffran, J. E. Treisman, and E. Y. Skolnik, 2000 The Ste20 kinase misshapen regulates both photoreceptor axon targeting and dorsal closure, acting downstream of distinct signals. Mol Cell Biol 20: 4736–4744.
25. Chaki, S. P., R. Barhoumi, and G. M. Rivera, 2019 Nck adapter proteins promote podosome biogenesis facilitating extracellular matrix degradation and cancer invasion. Cancer Med 8: 7385–7398.
26. Dubrac, A., G. Genet, R. Ola, F. Zhang, L. Pibouin-Fragner *et al.*, 2016 Targeting NCK-Mediated Endothelial Cell Front-Rear Polarity Inhibits Neovascularization. Circulation 133: 409–421.
27. Ruusala, A., T. Pawson, C.-H. Heldin, and P. Aspenström, 2008 Nck adapters are involved in the formation of dorsal ruffles, cell migration, and Rho signaling downstream of the platelet-derived growth factor beta receptor. J Biol Chem 283: 30034–30044.
28. Klaus, J., S. Kanton, C. Kyrousi, A. C. Ayo-Martin, R. Di Giaimo *et al.*, 2019 Altered neuronal migratory trajectories in human cerebral organoids derived from individuals with neuronal heterotopia. Nat Med 25: 561–568.
29. Deshpande, R. P., M. Panigrahi, C. S. Y B V K, and P. P. Babu, 2019 Expression and clinicopathological significance of Nck1 in human astrocytoma progression. Int J Neurosci 129: 171–178.
30. Boyle, M. J., R. L. French, K. A. Cosand, J. B. Dorman, D. P. Kiehart *et al.*, 2010 Division of labor: subsets of dorsal-appendage-forming cells control the shape of the entire tube. Dev Biol 346: 68–79.
31. Ghosh, P., A. O. Beas, S. J. Bornheimer, M. Garcia-Marcos, E. P. Forry *et al.*, 2010 A G{alpha}i-GIV molecular complex binds epidermal growth factor receptor and determines whether cells migrate or proliferate. Mol Biol Cell 21: 2338–2354.
32. Li, J., S. Zhu, D. Kozono, K. Ng, D. Futalan *et al.*, 2014 Genome-wide shRNA screen revealed integrated mitogenic signaling between dopamine receptor D2 (DRD2) and epidermal growth factor receptor (EGFR) in glioblastoma. Oncotarget 5: 882–893.
33. Solis, G. P., O. Bilousov, A. Koval, A.-M. Lüchtenborg, C. Lin *et al.*, 2017 Golgi-Resident Gαo Promotes Protrusive Membrane Dynamics. Cell 170: 939-955.e24.
34. Wang, X., J. Bo, T. Bridges, K. D. Dugan, T. Pan *et al.*, 2006 Analysis of cell migration using whole-genome expression profiling of migratory cells in the Drosophila ovary. Dev Cell 10: 483–495.
35. Samarasekera, G. D. N. G., and V. J. Auld, 2018 C-terminal Src kinase (Csk) regulates the tricellular junction protein Gliotactin independent of Src. MBoC 29: 123–136.
36. Padash-Barmchi, M., K. Browne, K. Sturgeon, B. Jusiak, and V. J. Auld, 2010 Control of Gliotactin localization and levels by tyrosine phosphorylation and endocytosis is necessary for survival of polarized epithelia. J Cell Sci 123: 4052–4062.

37. Chandran, R. R., E. Iordanou, C. Ajja, M. Wille, and L. Jiang, 2014 Gene expression profiling of Drosophila tracheal fusion cells. Gene Expr Patterns 15: 112–123.
38. Llimargas, M., M. Strigini, M. Katidou, D. Karagogeos, and J. Casanova, 2004 Lachesin is a component of a septate junction-based mechanism that controls tube size and epithelial integrity in the Drosophila tracheal system. Development 131: 181–190.
39. Ariss, M. M., A. R. Terry, A. B. M. M. K. Islam, N. Hay, and M. V. Frolov, 2020 Amalgam regulates the receptor tyrosine kinase pathway through Sprouty in glial cell development in the Drosophila larval brain. J Cell Sci 133: jcs250837.
40. Bobyn, A., M. Zarrei, Y. Zhu, M. Hoffman, D. Brenner *et al.*, 2020 Ancestry and frequency of genetic variants in the general population are confounders in the characterization of germline variants linked to cancer. BMC Med Genet 21: 92.
41. Even, İ., İ. Akiva, and N. B. İyison, 2019 An in vivo RNAi mini-screen in Drosophila cancer models reveals novel potential Wnt targets in liver cancer. Turk J Gastroenterol 30: 198–207.
42. Chiaretti, S., and I. de Curtis, 2016 Role of Liprins in the Regulation of Tumor Cell Motility and Invasion. Curr Cancer Drug Targets 16: 238–248.
43. Sakamoto, S., S. Narumiya, and T. Ishizaki, 2012 A new role of multi scaffold protein Liprin-α: Liprin-α suppresses Rho-mDia mediated stress fiber formation. Bioarchitecture 2: 43–49.
44. Chiaretti, S., and I. de Curtis, 2016 Role of Liprins in the Regulation of Tumor Cell Motility and Invasion. Curr Cancer Drug Targets 16: 238–248.
45. Jakobsen, K. R., E. Sørensen, K. K. Brøndum, T. F. Daugaard, R. Thomsen *et al.*, 2013 Direct RNA sequencing mediated identification of mRNA localized in protrusions of human MDA-MB-231 metastatic breast cancer cells. J Mol Signal 8: 9.
46. Li, F., W. Zhang, M. Wang, and P. Jia, 2020 IL1RAP regulated by PRPRD promotes gliomas progression via inducing neuronal synapse development and neuron differentiation in vitro. Pathol Res Pract 216: 153141.
47. Zou, J., X. Zhu, D. Xiang, Y. Zhang, J. Li *et al.*, 2021 LIX1-like protein promotes liver cancer progression via miR-21-3p-mediated inhibition of fructose-1,6-bisphosphatase. Acta Pharm Sin B 11: 1578–1591.
48. Xu, Y., C. Miao, C. Jin, C. Qiu, Y. Li *et al.*, 2018 SUSD2 promotes cancer metastasis and confers cisplatin resistance in high grade serous ovarian cancer. Exp Cell Res 363: 160–170.
49. Sang, J., X. Li, L. Lv, C. Zhang, X. Zhang *et al.*, 2021 Circ-TOP2A acts as a ceRNA for miR-346 and contributes to glioma progression via the modulation of sushi domain-containing 2. Mol Med Rep 23: 255.
50. Bravou, V., A. Antonacopoulou, S. Papanikolaou, S. Nikou, I. Lilis *et al.*, 2015 Focal Adhesion Proteins α- and β-Parvin are Overexpressed in Human Colorectal Cancer and Correlate with Tumor Progression. Cancer Invest 33: 387–397.
51. Johnstone, C. N., P. S. Mongroo, A. S. Rich, M. Schupp, M. J. Bowser *et al.*, 2008 Parvin-beta inhibits breast cancer tumorigenicity and promotes CDK9-mediated peroxisome proliferator-activated receptor gamma 1 phosphorylation. Mol Cell Biol 28: 687–704.
52. Tamir-Livne, Y., R. Mubariki, and E. Bengal, 2017 Adhesion molecule Kirrel3/Neph2 is required for the elongated shape of myocytes during skeletal muscle differentiation. Int J Dev Biol 61: 337–345.
53. Johnson, R. I., A. Sedgwick, C. D'Souza-Schorey, and R. L. Cagan, 2011 Role for a Cindr-Arf6 axis in patterning emerging epithelia. Mol Biol Cell 22: 4513–4526.
54. Hildebrand, J. D., A. D. Leventry, O. P. Aideyman, J. C. Majewski, J. A. Haddad *et al.*, 2021 A modifier screen identifies regulators of cytoskeletal architecture as mediators of Shroom-dependent changes in tissue morphology. Biol Open 10: bio055640.

55. Siang, L. C., R. Fernandez-Gonzalez, and J. J. Feng, 2018 Modeling cell intercalation during Drosophila germband extension. Phys Biol 15: 066008.
56. Haigo, S. L., J. D. Hildebrand, R. M. Harland, and J. B. Wallingford, 2003 Shroom induces apical constriction and is required for hingepoint formation during neural tube closure. Curr Biol 13: 2125–2137.
57. Razzell, W., M. E. Bustillo, and J. A. Zallen, 2018 The force-sensitive protein Ajuba regulates cell adhesion during epithelial morphogenesis. J Cell Biol 217: 3715–3730.
58. Buchert, M., M. Papin, C. Bonnans, C. Darido, W. S. Raye *et al.*, 2010 Symplekin promotes tumorigenicity by up-regulating claudin-2 expression. Proc Natl Acad Sci U S A 107: 2628–2633.
59. Bayer, C. A., S. R. Halsell, J. W. Fristrom, D. P. Kiehart, and L. von Kalm, 2003 Genetic interactions between the RhoA and Stubble-stubbloid loci suggest a role for a type II transmembrane serine protease in intracellular signaling during Drosophila imaginal disc morphogenesis. Genetics 165: 1417–1432.
60. Miao, W., N. Li, B. Gu, G. Yi, Z. Su *et al.*, 2020 LncRNA DLGAP1-AS2 modulates glioma development by up-regulating YAP1 expression. J Biochem 167: 411–418.
61. Liu, L., X. Li, Y. Shi, and H. Chen, 2021 Long noncoding RNA DLGAP1-AS1 promotes the progression of glioma by regulating the miR-1297/EZH2 axis. Aging (Albany NY) 13: 12129–12142.
62. Harris, K. E., and S. K. Beckendorf, 2007 Different Wnt signals act through the Frizzled and RYK receptors during Drosophila salivary gland migration. Development 134: 2017–2025.
63. Ewen-Campen, B., T. Comyn, E. Vogt, and N. Perrimon, 2020 No Evidence that Wnt Ligands Are Required for Planar Cell Polarity in Drosophila. Cell Rep 32: 108121.
64. Cohen, E. D., M.-C. Mariol, R. M. H. Wallace, J. Weyers, Y. G. Kamberov *et al.*, 2002 DWnt4 regulates cell movement and focal adhesion kinase during Drosophila ovarian morphogenesis. Dev Cell 2: 437–448.
65. Moura, R. S., E. Carvalho-Correia, P. daMota, and J. Correia-Pinto, 2014 Canonical Wnt signaling activity in early stages of chick lung development. PLoS One 9: e112388.
66. Sun, J., F. Macabenta, Z. Akos, and A. Stathopoulos, 2020 Collective Migrations of Drosophila Embryonic Trunk and Caudal Mesoderm-Derived Muscle Precursor Cells. Genetics 215: 297–322.
67. Doren, M. V., and R. Lehmann, 1997 Cell migration: Don’t tread on me. Current Biology 7: R148–R150.
68. Haack, T., M. Schneider, B. Schwendele, and A. D. Renault, 2014 Drosophila heart cell movement to the midline occurs through both cell autonomous migration and dorsal closure. Dev Biol 396: 169–182.
