# Supplementary material for: A Drosophila RNAi screen reveals conserved glioblastoma-related adhesion genes that regulate collective cell migration
Source: G3 (Bethesda). 2021 Oct 11;12(1):jkab356. doi: 10.1093/g3journal/jkab356 (PMC8728034; doi:10.1093/g3journal/jkab356)
Supplement: jkab356_Supplementary_Figure5 [file jkab356_supplementary_figure5.pdf]

Supplementary File 2. List of references for Supplementary Table 1.

1. Mummery-Widmer, J. L., M. Yamazaki, T. Stoeger, M. Novatchkova, S. Bhalerao *et al.*, 2009 Genome-wide analysis of Notch signalling in Drosophila by transgenic RNAi. Nature 458: 987–992.
2. Syrzycka, M., G. Hallson, K. A. Fitzpatrick, I. Kim, S. Cotsworth *et al.*, 2019 Genetic and Molecular Analysis of Essential Genes in Centromeric Heterochromatin of the Left Arm of Chromosome 3 in Drosophila melanogaster. G3 (Bethesda) 9: 1581–1595.
3. Berns, N., I. Woichansky, S. Friedrichsen, N. Kraft, and V. Riechmann, 2014 A genome-scale in vivo RNAi analysis of epithelial development in Drosophila identifies new proliferation domains outside of the stem cell niche. Journal of Cell Science 127: 2736–2748.
4. Petzoldt, A. G., J.-B. Coutelis, C. Géminard, P. Spéder, M. Suzanne *et al.*, 2012 DE-Cadherin regulates unconventional Myosin ID and Myosin IC in Drosophila left-right asymmetry establishment. Development 139: 1874–1884.
5. González-Morales, N., C. Géminard, G. Lebreton, D. Cerezo, J.-B. Coutelis *et al.*, 2015 The Atypical Cadherin Dachsous Controls Left-Right Asymmetry in Drosophila. Dev Cell 33: 675–689.
6. Rauskolb, C., G. Pan, B. V. V. G. Reddy, H. Oh, and K. D. Irvine, 2011 Zyxin links fat signaling to the hippo pathway. PLoS Biol 9: e1000624.
7. Sarpal, R., M. Pellikka, R. R. Patel, F. Y. W. Hui, D. Godt *et al.*, 2012 Mutational analysis supports a core role for Drosophila α-catenin in adherens junction function. J Cell Sci 125: 233–245.
8. Yue, T., A. Tian, and J. Jiang, 2012 The cell adhesion molecule echinoid functions as a tumor suppressor and upstream regulator of the Hippo signaling pathway. Dev Cell 22: 255–267.
9. Mao, Y., B. Kucuk, and K. D. Irvine, 2009 Drosophila lowfat, a novel modulator of Fat signaling. Development 136: 3223–3233.
10. Destalminil-Letourneau, M., I. Morin-Poulard, Y. Tian, N. Vanzo, and M. Crozatier, 2021 The vascular niche controls Drosophila hematopoiesis via fibroblast growth factor signaling. Elife 10: e64672.
11. Hogan, J., M. Valentine, C. Cox, K. Doyle, and S. Collier, 2011 Two frizzled planar cell polarity signals in the Drosophila wing are differentially organized by the Fat/Dachsous pathway. PLoS Genet 7: e1001305.
12. Misra, J. R., and K. D. Irvine, 2016 Vamana Couples Fat Signaling to the Hippo Pathway. Dev Cell 39: 254–266.
13. Revilla-Yates, E., L. Varas, J. Sierra, and I. Rodriguez, 2015 Transcriptional analysis of the dachsous gene uncovers novel isoforms expressed during development in Drosophila. FEBS Lett 589: 3595–3603.
14. Ambegaonkar, A. A., G. Pan, M. Mani, Y. Feng, and K. D. Irvine, 2012 Propagation of Dachsous-Fat planar cell polarity. Curr Biol 22: 1302–1308.
15. Harrison, N. J., E. Connolly, A. Gascón Gubieda, Z. Yang, B. Altenhein *et al.*, 2021 Regenerative neurogenic response from glia requires insulin-driven neuron-glia communication. Elife 10: e58756.
16. Cho B, Song S, Axelrod JD. 2020. Prickle isoforms determine handedness of helical morphogenesis. Elife. 9:e51456. doi:10.7554/eLife.51456.
17. Zeng, X., L. Han, S. R. Singh, H. Liu, R. A. Neumüller *et al.*, 2015 Genome-wide RNAi Screen Identifies Networks Involved in Intestinal Stem Cell Regulation in Drosophila. Cell Reports 10: 1226–1238.

18. Wang, Y., V. F. Naturale, and P. N. Adler, 2017 Planar Cell Polarity Effector Fritz Interacts with Dishevelled and Has Multiple Functions in Regulating PCP. G3 (Bethesda) 7: 1323–1337.
19. Tutor, A. S., S. Prieto-Sánchez, and M. Ruiz-Gómez, 2014 Src64B phosphorylates Dumbfounded and regulates slit diaphragm dynamics: Drosophila as a model to study nephropathies. Development 141: 367–376.
20. Gao, Y., Y. Mao, R.-G. Xu, R. Zhu, M. Zhang *et al.*, 2019 Defining gene networks controlling the maintenance and function of the differentiation niche by an in vivo systematic RNAi screen. J Genet Genomics 46: 19–30.
21. Kamiyama, D., R. McGorty, R. Kamiyama, M. D. Kim, A. Chiba *et al.*, 2015 Specification of Dendritogenesis Site in Drosophila aCC Motoneuron by Membrane Enrichment of Pak1 through Dscam1. Dev Cell 35: 93–106.
22. Gao, Y., Y. Mao, R.-G. Xu, R. Zhu, M. Zhang *et al.*, 2019 Defining gene networks controlling the maintenance and function of the differentiation niche by an in vivo systematic RNAi screen. J Genet Genomics 46: 19–30.
23. Poon, C. L. C., J. I. Lin, X. Zhang, and K. F. Harvey, 2011 The sterile 20-like kinase Tao-1 controls tissue growth by regulating the Salvador-Warts-Hippo pathway. Dev Cell 21: 896–906.
24. Swaminathan, A., V. L. Barnes, S. Fox, S. Gammouh, and L. A. Pile, 2012 Identification of genetic suppressors of the Sin3A knockdown wing phenotype. PLoS One 7: e49563.
25. Dye, N. A., M. Popović, K. V. Iyer, J. F. Fuhrmann, R. Piscitello-Gómez *et al.*, 2021 Self-organized patterning of cell morphology via mechanosensitive feedback. Elife 10: e57964.
26. Misra, J. R., and K. D. Irvine, 2019 Early girl is a novel component of the Fat signaling pathway. PLoS Genet 15: e1007955.
27. Pan, G., Y. Feng, A. A. Ambegaonkar, G. Sun, M. Huff *et al.*, 2013 Signal transduction by the Fat cytoplasmic domain. Development 140: 831–842.
28. Huang, H.-L., S. Wang, M.-X. Yin, L. Dong, C. Wang *et al.*, 2013 Par-1 regulates tissue growth by influencing hippo phosphorylation status and hippo-salvador association. PLoS Biol 11: e1001620.
29. Nagai, H., H. Tatara, K. Tanaka-Furuhashi, S. Kurata, and T. Yano, 2021 Homeostatic Regulation of ROS-Triggered Hippo-Yki Pathway via Autophagic Clearance of Ref(2)P/p62 in the Drosophila Intestine. Dev Cell 56: 81-94.e10.
30. Ke, H., Z. Feng, M. Liu, T. Sun, J. Dai *et al.*, 2018 Collagen secretion screening in Drosophila supports a common secretory machinery and multiple Rab requirements. J Genet Genomics S1673-8527(18)30097–3.
31. Oh, H., and K. D. Irvine, 2008 In vivo regulation of Yorkie phosphorylation and localization. Development 135: 1081–1088.
32. Koon, A. C., and V. Budnik, 2012 Inhibitory control of synaptic and behavioral plasticity by octopaminergic signaling. J Neurosci 32: 6312–6322.
33. Ignatious Raja, J. S., N. Katanayeva, V. L. Katanaev, and C. G. Galizia, 2014 Role of Go/i subgroup of G proteins in olfactory signaling of Drosophila melanogaster. Eur J Neurosci 39: 1245–1255.
34. Bredendiek, N., J. Hütte, A. Steingräber, H. Hatt, G. Gisselmann *et al.*, 2011 Go α is involved in sugar perception in Drosophila. Chem Senses 36: 69–81.
35. Agrawal, T., and G. Hasan, 2015 Maturation of a central brain flight circuit in Drosophila requires Fz2/Ca^{2+} signaling. Elife 4.
36. Qin, B., T.-H. Humberg, A. Kim, H. S. Kim, J. Short *et al.*, 2019 Muscarinic acetylcholine receptor signaling generates OFF selectivity in a simple visual circuit. Nat Commun 10: 4093.
37. Yan, D., R. A. Neumüller, M. Buckner, K. Ayers, H. Li *et al.*, 2014 A regulatory network of Drosophila germline stem cell self-renewal. Dev Cell 28: 459–473.

38. Neely, G. G., A. Hess, M. Costigan, A. C. Keene, S. Goulas *et al.*, 2010 A genome-wide Drosophila screen for heat nociception identifies α2δ3 as an evolutionarily conserved pain gene. Cell 143: 628–638.
39. Czech, B., J. B. Preall, J. McGinn, and G. J. Hannon, 2013 A transcriptome-wide RNAi screen in the Drosophila ovary reveals factors of the germline piRNA pathway. Mol Cell 50: 749–761.
40. Zhou, M., N. Chen, J. Tian, J. Zeng, Y. Zhang *et al.*, 2019 Suppression of GABAergic neurons through D2-like receptor secures efficient conditioning in Drosophila aversive olfactory learning. Proc Natl Acad Sci U S A 116: 5118–5125.
41. Solis, G. P., O. Bilousov, A. Koval, A.-M. Lüchtenborg, C. Lin *et al.*, 2017 Golgi-Resident Gαo Promotes Protrusive Membrane Dynamics. Cell 170: 939-955.e24.
42. Lin, C., and V. L. Katanaev, 2013 Kermit interacts with Gαo, Vang, and motor proteins in Drosophila planar cell polarity. PLoS One 8: e76885.
43. Hoxha, V., C. Lama, P. L. Chang, S. Saurabh, N. Patel *et al.*, 2013 Sex-specific signaling in the blood-brain barrier is required for male courtship in Drosophila. PLoS Genet 9: e1003217.
44. Cho, S., A. K. Muthukumar, T. Stork, J. C. Coutinho-Budd, and M. R. Freeman, 2018 Focal adhesion molecules regulate astrocyte morphology and glutamate transporters to suppress seizure-like behavior. Proc Natl Acad Sci U S A 115: 11316–11321.
45. Even, İ., İ. Akiva, and N. B. İyison, 2019 An in vivo RNAi mini-screen in Drosophila cancer models reveals novel potential Wnt targets in liver cancer. Turk J Gastroenterol 30: 198–207.
46. Urwyler, O., A. Izadifar, D. Dascenco, M. Petrovic, H. He *et al.*, 2015 Investigating CNS synaptogenesis at single-synapse resolution by combining reverse genetics with correlative light and electron microscopy. Development 142: 394–405.
47. Valakh, V., S. A. Naylor, D. S. Berns, and A. DiAntonio, 2012 A large-scale RNAi screen identifies functional classes of genes shaping synaptic development and maintenance. Dev Biol 366: 163–171.
48. Vakaloglou, K. M., M. Chountala, and C. G. Zervas, 2012 Functional analysis of parvin and different modes of IPP-complex assembly at integrin sites during Drosophila development. J Cell Sci 125: 3221–3232.
49. Takemura, M., and T. Adachi-Yamada, 2011 Cell death and selective adhesion reorganize the dorsoventral boundary for zigzag patterning of Drosophila wing margin hairs. Dev Biol 357: 336–346.
50. Sugie, A., D. Umetsu, T. Yasugi, K.-F. Fischbach, and T. Tabata, 2010 Recognition of pre- and postsynaptic neurons via nephrin/NEPH1 homologs is a basis for the formation of the Drosophila retinotopic map. Development 137: 3303–3313.
51. Langer, C. C. H., R. K. Ejsmont, C. Schönbauer, F. Schnorrer, and P. Tomancak, 2010 In vivo RNAi rescue in Drosophila melanogaster with genomic transgenes from Drosophila pseudoobscura. PLoS One 5: e8928.
52. Kuckwa, J., K. Fritzen, D. Buttgereit, S. Rothenbusch-Fender, and R. Renkawitz-Pohl, 2016 A new level of plasticity: Drosophila smooth-like testes muscles compensate failure of myoblast fusion. Development 143: 329–338.
53. Johnson, R. I., A. Sedgwick, C. D'Souza-Schorey, and R. L. Cagan, 2011 Role for a Cindr-Arf6 axis in patterning emerging epithelia. Mol Biol Cell 22: 4513–4526.
54. Schnorrer, F., C. Schönbauer, C. C. H. Langer, G. Dietzl, M. Novatchkova *et al.*, 2010 Systematic genetic analysis of muscle morphogenesis and function in Drosophila. Nature 464: 287–291.
55. Ansar, M., H.-L. Chung, A. Al-Otaibi, M. N. Elagabani, T. A. Ravenscroft *et al.*, 2019 Bi-allelic Variants in IQSEC1 Cause Intellectual Disability, Developmental Delay, and Short Stature. Am J Hum Genet 105: 907–920.

56. Hildebrand, J. D., A. D. Leventry, O. P. Aideyman, J. C. Majewski, J. A. Haddad *et al.*, 2021 A modifier screen identifies regulators of cytoskeletal architecture as mediators of Shroom-dependent changes in tissue morphology. Biol Open 10: bio055640.
57. Shan, L., C. Wu, D. Chen, L. Hou, X. Li *et al.*, 2017 Regulators of alternative polyadenylation operate at the transition from mitosis to meiosis. J Genet Genomics 44: 95–106.
58. Marcogliese, P. C., S. Abuaish, G. Kabbach, E. Abdel-Messih, S. Seang *et al.*, 2017 LRRK2(I2020T) functional genetic interactors that modify eye degeneration and dopaminergic cell loss in Drosophila. Hum Mol Genet 26: 1247–1257.
59. Chen, Z., J.-Y. Zhu, Y. Fu, A. Richman, and Z. Han, 2016 Wnt4 is required for ostia development in the Drosophila heart. Dev Biol 413: 188–198.
60. Ewen-Campen, B., T. Comyn, E. Vogt, and N. Perrimon, 2020 No Evidence that Wnt Ligands Are Required for Planar Cell Polarity in Drosophila. Cell Rep 32: 108121.
61. Mottier-Pavie, V. I., V. Palacios, S. Eliazer, S. Scoggin, and M. Buszczak, 2016 The Wnt pathway limits BMP signaling outside of the germline stem cell niche in Drosophila ovaries. Dev Biol 417: 50–62.
62. Stepanik, V., L. Dunipace, Y.-K. Bae, F. Macabenta, J. Sun *et al.*, 2016 The migrations of Drosophila muscle founders and primordial germ cells are interdependent. Development 143: 3206–3215.
63. Kumar, S., I.-H. Jang, C. W. Kim, D.-W. Kang, W. J. Lee *et al.*, 2016 Functional screening of mammalian mechanosensitive genes using Drosophila RNAi library- Smarcd3/Bap60 is a mechanosensitive pro-inflammatory gene. Sci Rep 6: 36461.
64. Pantoja, M., K. A. Fischer, N. Ieronimakis, M. Reyes, and H. Ruohola-Baker, 2013 Genetic elevation of sphingosine 1-phosphate suppresses dystrophic muscle phenotypes in Drosophila. Development 140: 136–146.
65. Chabu, C., D.-M. Li, and T. Xu, 2017 EGFR/ARF6 regulation of Hh signalling stimulates oncogenic Ras tumour overgrowth. Nat Commun 8: 14688.
66. Ben-Zvi, D. S., and T. Volk, 2019 Escort cell encapsulation of Drosophila germline cells is maintained by irre cell recognition module proteins. Biol Open 8.
67. Sauerwald, J., W. Backer, T. Matzat, F. Schnorrer, and S. Luschnig, 2019 Matrix metalloproteinase 1 modulates invasive behavior of tracheal branches during entry into Drosophila flight muscles. Elife 8: e48857.
