# Supplementary material for: A Drosophila RNAi screen reveals conserved glioblastoma-related adhesion genes that regulate collective cell migration
Source: G3 (Bethesda). 2021 Oct 11;12(1):jkab356. doi: 10.1093/g3journal/jkab356 (PMC8728034; doi:10.1093/g3journal/jkab356)
Supplement: jkab356_Supplementary_Table1 [file jkab356_supplementary_table1.pdf]

Supplementary Table 1. RNAi lines from screen that have phenotypes in other systems in *Drosophila*.

Gene	RNAi	Stock center	Construct ID	Other systems
**alpha-catenin (α-cat)**	20123	VDRC	GD8808	Pupal lethal under pnr-GAL4 and lethal under tubulin driver[1,2]
	40882	VDRC	GD8808	Pupal lethal under pnr-GAL4[1]; rotation defects in male genitalia[4]; epithelial defects in the ovaries when driven by *traffic jam*-Gal4[7]
	107298	VDRC	KK107916	Epithelial defects in the ovaries when driven by *escargot (esg)*-GAL4 and *traffic jam*-Gal4[3,7]; rotation defects in male genitalia[4]
**Dachsous (ds)**	36219	VDRC	GD14350	Genome-wide bristle screen[1]; larval hindgut in LR asymmetry[5]; size defect, wing imaginal disc and adult wing[6,8,9,13]; cardiac cells[10]; wing shape and local hair polarity[11]; lethal under esg-GAL4[17]
	4313	VDRC	GD2646	Adult wing and wing disc abnormality[12,13,14]; larval hindgut in LR asymmetry[5]; reduced escort cell extension[66]
	32964	BL	HMS00759	Larval ventral nerve cord[15]; anterior wing margin bristle apical rotation[16], abdomen PCP[18]; lethal under Act5C-GAL4[17]; larval/pupal lethal and flight muscle defect under c587 and nos-GAL4s[20,67]
**Dreadlocks (dock)**	37524	VDRC	GD4034	Agglutination of larval nephrocytes[19]
	27728	BL	JF02810	Embryonic CNS (anterior corner cell) and adult CNS[21]; lethal under Act5C-GAL4, nos-GAL4 and c587-GAL4[17,20]
**Fat**	108863	VDRC	KK101190	Larval hindgut in LR asymmetry[5]; abnormal adult wing and wing disc[23,24]; reduced escort cell extension[66]
	9396	VDRC	GD881	Genome-wide bristle screen[1]; larval hindgut in LR asymmetry[5]; wing disc and adult wing[6,11,12,25,26,27,28]; enterocytes[29]; larval brain and eye disc[31]

Gene	RNAi	Stock center	Construct ID	Other systems
**G protein alpha i subunit**	40890	BL	HMS02138	ECM defects in 3rd instar larvae[30]
	28150	VDRC	GD12576	Genome-wide bristle screen[1]; fly antennae for olfaction[33]
**G protein alpha o subunit**	34653	BL	HMS01129	Larval and adult brain[35,36]; lethal under Act5C-GAL4 and MTD-GAL4[17,37]
	110552	VDRC	KK109018	Octopaminergic neurons[32]
	19124	VDRC	GD8640	Genome-wide bristle screen[1]; embryonic lethal under elav-GAL4[38]; Anterior Paired Lateral (APL) neurons and adult brain[40,32]; gustatory receptors[34]; wing and notum[41,42]; male courtship[43]
**Gliotactin**	37115	VDRC	GD1735	Genome-wide bristle screen[1]
	37116	VDRC	GD1735	Genome-wide bristle screen[1]
**Lachesin (Lac)**	35524	VDRC	GD12649	pupal lethal under pnr-GAL4[1]
	107450	VDRC	KK107469	Epithelial defects during oogenesis[3,39]; enhanced astrocyte seizure activity[44];
	28940	BL	HM05151	lethal under Act5C-GAL4[17]
**Liprin-alpha**	106588	VDRC	KK10116	Fly eye tumor model (overexpression of a Notch ligand leads to benign overgrowths in the eyes)[45]; adult mechanosensory neurons[46]; neuronal defects[47]
**Mesh**	6867	VDRC	GD3140	Pupal lethal under pnrGAL4[1]
**Parvin**	105356	VDRC	KK102567	Larval wing disc[49]; larval lethal under MEf2-GAL4[51]

Gene	RNAi	Stock center	Construct ID	Other systems
Roughest (rst)	27223	VDRC	GD14475	Genome-wide bristle screen[1], wing margin hair[49]
	27225	VDRC	GD14475	Larval optic lobe[50]
	951	VDRC	GD86	Genome-wide bristle screen[1], male genital disc and myoblasts around adult male genitalia[52]
Schizo	36625	VDRC	GD14895	Patterning of the eye[53]
	36627	VDRC	GD14895	Patterning of the eye[53;] flight defective[54]; suppression of larval eye disc tumors[65]
	106168	VDRC	KK103616	Neurons under elav-GAL4[55]; suppression of larval eye disc tumors[65]
	39060	BL	HMS01980	Neurons under elav-GAL4[55]; suppression of larval eye disc tumors[65]; pupal lethal under c587-GAL4[20]
Shroom	47147	VDRC	GD16363	Retinal degeneration[58]
	100672	VDRC	KK106863	Wing imaginal disc[56]
Symplekin (Sym)	33469	VDRC	GD9722	Pupal lethal under pnrGAL4[1]
	33470	VDRC	GD9722	Pupal lethal under pnrGAL4[1]; partial lethal under elav-GAL4 and lethal under Mef2-GAL4[38,54]
	39041	BL	HMS01961	Mitotic spermatogonia[57]
Vulcan	46230	VDRC	GD16319	Increased astrocyte seizure activity[44]
Wnt4	38011	VDRC	GD5347	Pain response defects[38]
	38010	VDRC	GD5347	Larval cardiac cells[10]

Gene	RNAi	Stock center	Construct ID	Other systems
	104671	VDRC	KK102348	Larval cardiac cells[10]; ostia progenitor cells[59]; egg chamber defects during oogenesis under traffic jam-GAL4[3]
	29442	BL	JF03378	Wing disc and adult wing[60]; germline stem cell defects in ovaries under c587-GAL4[61]
**Wunen**	6446	VDRC	GD1640	Caudal visceral mesoderm cell migration (weak phenotype)[62]; bacterial infection induced *drosomycin* expression[63]; suppression of dystrophic muscle phenotype[64]

References are in Supplementary File 2.
