# Supplementary material for: A Drosophila RNAi screen reveals conserved glioblastoma-related adhesion genes that regulate collective cell migration
Source: G3 (Bethesda). 2021 Oct 11;12(1):jkab356. doi: 10.1093/g3journal/jkab356 (PMC8728034; doi:10.1093/g3journal/jkab356)
Supplement: jkab356_Supplementary_Table2 [file jkab356_supplementary_table2.pdf]

Supplementary Table 2. Statistics for gene expression in Ivy GAP analyses.

SYPMK

	MP	HBV	PAN	PNZ	CT	IT
LE	0.999	>0.999	0.962	0.954	0.959	0.940
IT	0.735	0.940	>0.999	>0.999	0.999	-
CT	0.678	0.957	>0.999	>0.999	-	
PNZ	0.766	0.954	>0.999	-		
PAN	0.756	0.962	-			
HBV	0.999	-				

NCK1

	MP	HBV	PAN	PNZ	CT	IT
LE	***	**	***	*	0.484	0.980
IT	***	*	***	0.124	0.965	-
CT	***	0.050	***	0.189	-	
PNZ	0.309	0.997	**	-		
PAN	0.850	0.073	-			
HBV	0.737	-				

CTNNA1

	MP	HBV	PAN	PNZ	CT	IT
LE	***	***	***	***	***	***
IT	***	**	***	***	***	-
CT	0.814	0.998	***	0.534	-	
PNZ	0.999	0.959	0.589	-		
PAN	0.293	0.099	-			
HBV	0.996	-				

CTNNA2

	MP	HBV	PAN	PNZ	CT	IT
LE	***	***	***	***	***	0.232
IT	***	***	***	***	0.398	-
CT	***	***	***	**	-	
PNZ	*	0.368	>0.999	-		
PAN	*	0.388	-			
HBV	0.939	-				

CTNNA3

	MP	HBV	PAN	PNZ	CT	IT
LE	0.198	**	***	**	0.140	0.828
IT	0.931	0.157	*	0.103	0.955	-
CT	0.999	0.285	*	0.171	-	
PNZ	0.621	>0.999	>0.999	-		
PAN	0.368	>0.999	-			
HBV	0.716	-				

NEGR1

	MP	HBV	PAN	PNZ	CT	IT
LE	***	***	***	***	***	***
IT	***	***	***	***	***	-
CT	0.120	0.295	**	0.401	-	
PNZ	0.999	>0.999	0.950	-		
PAN	0.997	0.993	-			
HBV	>0.999	-				

DCHS1

	MP	HBV	PAN	PNZ	CT	IT
LE	**	**	**	***	***	0.063
IT	0.979	0.967	0.956	0.836	0.934	-
CT	>0.999	>0.999	>0.999	0.996	-	
PNZ	0.998	0.999	0.999	-		
PAN	>0.999	>0.999	-			
HBV	>0.999	-				

FAT4

	MP	HBV	PAN	PNZ	CT	IT
LE	***	**	0.976	0.988	>0.999	>0.999
IT	***	**	0.962	0.982	0.999	-
CT	***	***	0.973	0.993	-	
PNZ	***	***	>0.999	-		
PAN	***	***	-			
HBV	0.999	-				

KIRREL1

	MP	HBV	PAN	PNZ	CT	IT
LE	***	***	***	***	*	0.759
IT	***	***	***	***	0.670	-
CT	***	***	***	***	-	
PNZ	**	0.062	0.697	-		
PAN	***	***	-			
HBV	0.975	-				

KIRREL2

	MP	HBV	PAN	PNZ	CT	IT
LE	0.992	>0.999	0.844	*	0.465	>0.999
IT	0.972	>0.999	0.869	*	0.456	-
CT	*	0.292	0.996	0.360	-	
PNZ	***	*	0.271	-		
PAN	0.232	0.728	-			
HBV	0.996	-				

KIRREL3

	MP	HBV	PAN	PNZ	CT	IT
LE	***	***	***	***	***	***
IT	***	***	***	***	***	-
CT	***	**	0.371	0.085	-	
PNZ	0.921	0.988	0.975	-		
PAN	0.333	0.638	-			
HBV	>0.999	-				
