# Supplementary material for: A Drosophila RNAi screen reveals conserved glioblastoma-related adhesion genes that regulate collective cell migration
Source: G3 (Bethesda). 2021 Oct 11;12(1):jkab356. doi: 10.1093/g3journal/jkab356 (PMC8728034; doi:10.1093/g3journal/jkab356)
Supplement: jkab356_Supplementary_Figures-Tables-Captions [file jkab356_supplementary_figures-tables-captions.docx]

**Supplementary Figure 1.** Regional expression of human ortholog adhesion genes in GBM patient tumors for additional human genes. See Figure 4 legend for abbreviations of the tumor regions. Data from the Ivy GAP are shown as mean expression +/- SD across GBM tumor regions. Statistics are shown in Supplementary Table 2: *p<0.05; **p<0.01; ***p<0.001, one way ANOVA with Tukey HSD.

**Supplementary Figure 2.** Expression of human ortholog adhesion genes across glioma tumor grade. Box plots of mRNA expression obtained from the TCGA database in grade II (n=226), grade III (n=244), and grade IV (n=150) patient gliomas. *p<0.05; **p<0.01; ***p<0.001, one way ANOVA with Tukey HSD.

**Supplementary Figure 3.** Expression of human ortholog adhesion genes in GBM compared to non-tumor brain tissue. Box plots of mRNA expression obtained from the GEPIA database in non-tumor (n=207) and GBM tumor (n=163). *p<0.01.

**Supplementary Table 1.** List of *Drosophila* RNAi lines with various phenotypes in other studies.

**Supplementary Table 2.** Statistics for the Ivy GAP regional gene expression for all human adhesion gene orthologs. Graphed data are shown in Figure 4 and Supplementary Figure 1.

**Supplementary File 1.** List of references for Table 1.

**Supplementary File 2.** List of references for Supplementary Table 1.
